# Exposure to an anti-androgenic herbicide negatively impacts reproductive physiology and fertility in *Xenopus tropicalis*

**DOI:** 10.1038/s41598-018-27161-2

**Published:** 2018-06-14

**Authors:** F. Orton, M. Säfholm, E. Jansson, Y. Carlsson, A. Eriksson, J. Fick, T. Uren Webster, T. McMillan, M. Leishman, B. Verbruggen, T. Economou, C. R. Tyler, C. Berg

**Affiliations:** 1 School of Science and Sport, University of the West of Scotland, Paisley, UK; 2 Department of Environmental Toxicology, Uppsala University, Uppsala, Sweden; 3 Department of Chemistry, Umeå University, Umeå, Sweden; 4 Swansea University, Swansea, UK; 5 College of Life and Environmental Sciences, University of Exeter, Exeter, UK

## Abstract

Amphibians are threatened on a global scale and pollutants may be contributing to population declines, but how chemicals impact on their reproduction is poorly understood. We conducted a life cycle analysis to investigate the impacts of early life exposure to two anti-androgens (exposure until completion of metamorphosis;stage 66): flutamide, (50 µg/L)/linuron (9 and 45 µg/L)) on sexual development and breeding competence in *Xenopus tropicalis*. Our analyses included: mRNA levels of *dmrt1, cyp17, amh, cyp19, foxl2* and *ar* (tadpoles/metamorphs), gonadal histomorphology (metamorphs/adults), mRNA levels of *ar*/*gr* (adult male brain/gonad/forelimb), testosterone/corticosterone levels (adult males), secondary sexual characteristics (forelimb width/nuptial pad: adult males) and breeding competence (amplexus/fertility: adult males). Compared to controls, feminised sex ratios and increased number of spermatogonia (adults) were observed after exposure to flutamide and the lower linuron concentration. Exposure to the lower linuron concentration also resulted in demasculinisation of secondary sexual characteristics and reduced male fertility. Flutamide exposure resulted in masculinisation of the nuptial pad and elevated mRNA levels of *dmrt1*, *cyp17*, *amh* and *foxl2* in brains (metamorphs). Testosterone levels were higher in all treatment groups, however, overall few effects were observed in response to the higher linuron concentration. Our findings advance understanding of reproductive biology of *X. tropicalis* and illustrate negative effects of linuron on reproductive processes at a concentration measured in freshwater environments.

## Introduction

Globally, amphibian populations are disappearing at a rate faster than for any other vertebrate group^1^. Recently it was reported that as many as 40% of known amphibian species face imminent extinction^2^. Overall, habitat destruction and fragmentation, are probably the main causes of amphibian decline^3^, however, introductions of foreign species, climate change, pollution and disease may also be significant contributing factors. Various chemicals associated with agricultural intensification and with effluent discharges have been shown to impact adversely on individual amphibian health^4–6^ and many of these chemicals have been shown to exert their effects via the endocrine system, affecting sex and reproduction^7,8^. Exposure to these so-called endocrine disrupting chemicals (EDCs) at critical periods during early life has been shown to cause subsequent effects in adult frogs, including gonadal sex-reversal, reduced gametogenesis and impaired fertility^9,10^, but identification of associated mechanisms remains elusive. The lack of markers of genetic sex in amphibians (except for *X. laevis*^11^) has made it especially difficult to assess for sex-specific effects of EDCs during early life stages, prior to gonadal differentiation.

In the absence of sex-specific genetic markers, a possible approach to identify sex is use of the expression of genes that are sexually dimorphic. Examples of genes that show sexually dimorphic expression in amphibians include cytochrome P450 aromatase (*cyp19*: converts androgens to estrogens), cytochrome 17α-hydroxylase/17,20 lyase (*cyp17*: converts progestogens to androgens) and forkhead box protein L2 (*foxl2*: a transcription factor). *Cyp19* and *foxl2* are expressed at higher levels in female gonads compared with male gonads in *Lithobates sylvatica*, *X. tropicalis*^12^ ((Nieuwkoop-Faber (NF)^13^) developmental stage 50 in *X. tropicalis*; (Gosner (G)^14^) stage 30 in *L. sylvatica*) and in *Rana rugosa* (G stage 25^15^). In contrast, in *R. rugosa*, *cyp17* is expressed at a higher level in male gonads compared to females (G stage 25^16^). Levels of doublesex and mab-3 related transcription factor 1 *(dmrt1*: *R. rugosa*^17^) and anti-Müllerian hormone (*amh*: *Pleurodeles waltl*^18^, *X. tropicalis*^19^) mRNA are also higher in male gonads compared to female gonads during the period of sexual differentiation. The protein products DMRT1 and AMH have also been reported to occur at higher levels in the developing testes compared with ovarian tissue in several anuran species (*X. laevis, Bombina bombina, Bufo viridis, Hyla arborea, Rana arvalis, Rana temporaria*^20^). Using a knockout experimental system it has been reproted that the androgen receptor (*ar*) is essential for sex determination in *R. rugosa*^21^, although *ar* expression is not sexually dimorphic during gonadal differentiation in this species^22,23^.

Anti-androgenic chemicals are the most commonly occurring type of EDC and they have been associated with altered sexual development in fish^24,25^ and humans^26^. Many pesticides, including herbicides, possess anti-androgenic activity^27,28^ and high level application of agrochemicals occurs most often during the spring, coinciding with amphibian breeding and the period of sexual development during early life. Adult exposure to the anti-androgenic pesticide vinclozolin has been shown to reduce the prominence of nuptial (“thumb”) pads (an androgen dependent secondary sex feature^29^) in adult *X. laevis*^30^, although the breeding consequences of this effect are not known. In the UK, anti-androgenic activity has been reported widely in ponds containing breeding amphibians, although the chemicals responsible for this activity have not been characterised^31^. In laboratory studies some pharmaceuticals have been shown to be anti-androgenic, including cyproterone acetate^32^ and flutamide^33^ and for high level exposures they cause gonadal feminisation in amphibians. Other chemicals classified as anti-androgens, for example, the plasticiser di-n-butyl phthalate, can disrupt spermatogenesis in adult frogs following developmental exposure (35 µg/L)^34^. However, data on the effects of most environmentally relevant anti-androgens are lacking. Here, we tested an environmentally relevant concentration of the herbicide linuron (9 µg/L [32 nM])^35^ and a higher concentration (45 µg/L [181 nM]), to reflect peak concentrations that may occur in the environment as modelled by the U.S. Environmental Protection Agency^36^. In addition, we used flutamide (50 µg/L [181 nM]), a pharmaceutical androgen receptor antagonist, as a positive control for anti-androgenic effects.

There are few life-cycle studies with amphibians (but see^9,37,38^) and there are no reported studies for reproductive effects in adults arising from developmental exposure to anti-androgens in any non-mammalian vertebrate. Here we conducted a life cycle analysis with *X. tropicalis* exposed to the anti-androgens linuron and flutamide to assess for effects on sexual development (including gonadal differentiation and maturation), secondary sex characters, and subsequent gametogenesis, fertility and breeding behaviour in adult male and female frogs. We hypothesised that anti-androgenic chemicals would have feminising effects on physiological features, expression of gene related to sex in the brain and gonad (*dmrt1*, *amh, cyp17, ar, foxl2* and *cyp19*) and on behavioural endpoints in male frogs. We sought also to establish whether this gene set could be used to distinguish sex during early life prior to when gonadal sex was histologically distinguishable. As part of these analyses we furthermore sought to investigate for any relationships between breeding outcomes and secondary sex characteristics. *X. tropicalis* is an established model for investigating developmental reproductive toxicity^37^. It is water-dwelling throughout life, has a short generation time (4–6 months) and exposures to various chemicals with endocrine action have been shown to cause developmental reproductive toxicity in this species^9,37–40^. The sensitivity of gonadal differentiation to estrogenic endocrine disruption in *X. tropicalis* is comparable with that of a temperate, terrestrial frog species^41^.

## Methods

### Experimental Approach

**Figure 1:**
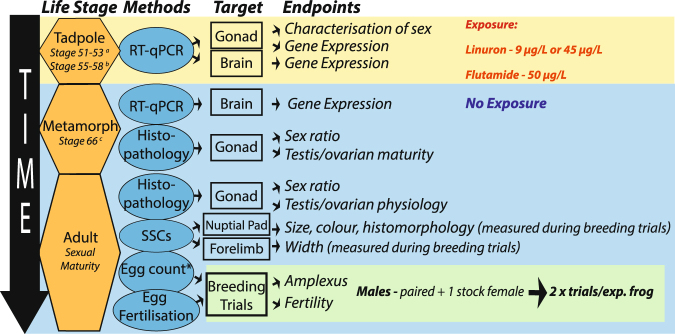
Experimental design. Fertilised eggs were exposed to flutamide or linuron until life stage 66 when they were transferred to clean water. Gene expression analysis on a set of target genes was conducted to identify their utility for distinguishing gonadal sex and to investigate the effects of anti-androgens on their expression in developing tadpoles (during sensitive windows of sexual development). Effects of anti-androgens on gonadal development and sex ratio were assessed in metamorphs via histopathology. Frogs were then maintained to adulthood in clean water when they were assessed for reproductive competence and morphology. Breeding studies were conducted to assess the effects of early life exposure to anti-androgens on the gonadal sexual development, secondary sex characters in males (nuptial pads and forearm) and breeding success (fecundity, fertility and time in amplexus). a=sex determination, b=sex differentiation, c=completed sex differentiation. SSC=secondary sexual characteristic, exp.=experimental.

Figure 1 provides a schematic illustration of the overall experimental approach. *X. tropicalis* were exposed from embryo NF stage 40 throughout the larval period to linuron (9 µg/L [32 nM] or 45 µg/L [181 nM]) or flutamide (50 µg/L [181 nM]) using a semi-static experimental design. Flutamide and linuron were analysed using gas chromatography/mass spectroscopy (GCMS) throughout the exposure period (for details of exposure conditions see Supplementary methods S1 and for animal husbandry see Supplementary methods S2). Survival and time to metamorphosis were recorded and tadpoles were sub-sampled at two time points, relating to sex determination (NF stages 51–53) and sex differentiation (NF stages 55–58)^42,43^. Tadpoles were sacrificed by pithing under anaesthesia (iced 1% tricaine methanesulfonate, pH 7.5). The stage of tadpole development was recorded for each animal, and the gonad-mesonephros complex (GMC) and brain were excised and placed in RNAlater (Sigma-Aldrich, USA) for analysis of gene expression (qRT-PCR; *dmrt1*, *amh, cyp17, ar, foxl2*, *cyp19*). The ability of this gene set for distinguishing sex in *X. tropicalis* in controls during early life, prior to when the gonad could be sexed by histology, was investigated, as well as the effects of chemical treatments on their ontogeny in both ‘males’ and ‘females’. At completion of metamorphosis (NF stage 66), exposure ceased and individuals were placed in new tanks containing test substance-free water (32 per tank, flow-through). Metamorphs (NF 66) not required for this grow out phase were sacrificed (pithing, 3% tricaine methanesulfonate, pH 7.5), the GMC collected and placed in neutral buffered formalin (NBF) for analysis of gonadal histomorphology (all histology analyses were undertaken without knowledge of exposure group) and the brains dissected and placed in RNAlater for analysis of gene expression. At 4 months post-metamorphosis, juvenile male/female frogs were placed in separate tanks to eliminate the possibility of non-controlled breeding. The effects of treatment on sex ratio, sex organ weights, gonadal histomorphology, breeding behaviour, fertility and on secondary sexual characteristics (nuptial pad size/colour/histomorphology and forelimb width in males) were investigated in sexually mature frogs (6 months post-metamorphosis). In adult females, ovarian histomorphology was analysed in a sub-sample that were sacrificed prior to breeding and in adult males, gonadal histomorphology was conducted on a sub-sample of individuals immediately following breeding. Adults were sacrificed by decapitation under anaesthesia (3% tricaine methanesulfonate, pH 7.5, 2 mins), blood was taken and analysed for testosterone and corticosterone levels and arm/brain/gonad were placed in RNAlater for analysis of gene expression (*gr*/*ar*/*rpl8*). The effects of treatments on reproductive morphology and breeding outcomes were investigated.

### Gonadal Histomorphology

Gonadal tissue for histological analysis was embedded in hydroxyethyl methacrylate (Technovit 7100, Heraeus Kulzer, Wehrheim, Germany) and all samples were sectioned (2 µm) and stained with haematoxylin-eosin for analysis using light microscopy (Leitz, Laborlux 12, Leica AB, Kista, see Supplementary text S3). In metamorphs, three successive transverse sections of the GMC were taken from the cranial end of the gonad. One section from each individual was taken randomly for examination and identified as either ovary (ovarian cavity surrounded by a cortex) or testis (medulla and lacking ovarian cavity). Ovaries were classified according to the most mature cell type observed in each section: oogonia, leptotene/pachytene or diplotene oocyte^37^. Testes were classified according to the stage of the most advanced germ cell and presence of primordial tubules: Stage 1 – primary spermatogonia, no tubules; Stage 2/3 – early secondary spermatogonia/late secondary spermatogonia with or without tubules^44^. In adult females, ovarian tissue (1–2 g) was excised from individuals from the same central region of the gonad that we have shown previously to be representative of the entire ovary in *X. tropicalis*^45^. One ovarian section per individual was taken at random for analysis. Oocytes were staged as pre-vitellogenic (stage I/II - no yolk granules), vitellogenic (stage III/V – yolk granules), post-vitellogenic (stage VI – unpigmented equatorial belt separating the animal and vegetal hemisphere) or atretic (segmented with vacuolization). The proportion of each stage was calculated compared to the total number of oocytes. In adult males, evaluation of testis histomorphology comprised of an analysis of germ cell types (spermatogonia, spermatocytes, spermatozoa) and testis structural features (cell nests, tubules) in 10 randomly taken individuals per treatment (see Supplementary methods S3).

### Nuptial Pad Histomorphology

Forelimb tissue for histological analysis was embedded in paraffin or hydroxyethyl methacrylate (Technovit 7100, Heraeus Kulzer, Wehrheim, Germany) and all samples were sectioned (5 µm) and stained with haematoxylin-eosin for analysis using light microscopy (Novex B-range). Transverse sections were cut along the entire length of the arm and sections at one quarter, half way, and three quarters of the way through the tissue were selected for analysis. The total number of breeding glands (glands containing mucous, staining pink) and keratinised hooks (protruding hook features) from the 3 selected sections were counted. See Supplementary Fig. S9, histomicrographs of analysed features.

### Gene Expression Analysis

Tissue samples (brain or GMC) were homogenized and lysed, RNA was extracted/isolated and cDNA synthesised. For tadpole/metamorph samples, a cDNA sample from pooled brain/GMC tissue was serially diluted and used as an internal standard in each PCR run. Internal standards covered ~10 cycles and all experimental samples were diluted to fall within the range of the standard curve. For the adult male arm, brain and gonad samples gene expression data was normalised using *rpl8* housekeeping gene. Each duplicate cDNA sample was tested in two independent PCR runs (in duplicate) and a no-template control (NTC) sample with water instead of cDNA was always included. Only data obtained from PCR runs with efficiency of 85–115 and r^2^ > 0.9, as well as no overlap between samples and NTCs were used (4 runs were excluded). See Supplementary methods S4 and Supplementary table S1 for details of primer design/sequence and PCR conditions.

### Plasma hormone analysis

Following euthanasia, blood was collected in heparinized capillary tubes by cardiac puncture and centrifuged (5 minutes, 1000 g). Plasma was snap frozen in liquid nitrogen. Upon thawing, plasma was extracted twice with ethyl acetate (each time equal volumes of ethylacetate:plasma), evaporated using nitrogen gas, and resuspended in twice the volume of ethanol than plasma, for use in assays (extraction efficiency: testosterone – 98%, corticosterone – 95%). The final dilution for assays was x200 for corticosterone and for testosterone/dihydrotestosterone. Corticosterone was quantified using radioimmunoassay (polyclonal antibody from Abcam, cross-reactivity <1.5%) and testosterone/dihydrotestosterone (equal binding affinity to the antibody) with enzyme-linked immunoassay (Oxford Biomedical Research, cross-reactivity <1%). All samples were run in duplicate within each assay. Intra-assay variability for corticosterone was 9.3% and for dihydrotestosterone/ testosterone was 6.9%.

### Breeding Trials and Secondary Sexual Characteristics

Breeding of male *X. tropicalis* took place 6 months post-metamorphosis using a competitive breeding system, whereby 2 experimental males were placed with 1 unexposed female. Two breeding trials were undertaken with each experimental male, ensuring a similar recovery time between breeding trials (minimum of 10 days). In total 158 trials were undertaken (92 trials for the first breeding, and 66 for the second breeding). To induce breeding, male and female frogs were given two injections of hCG: priming (20 IU) and boosting (23 hours later, 100 IU). Following the hCG boosting injection, each set of two males was placed in a breeding tank (20 L, darkened with plastic covering, containing 3 glass petri dishes), and a pre-weighed female was introduced. After 60 minutes, and thereafter every 45 minutes, tanks were assessed to identify the individual male in amplexus and whether spawning had occurred. See supplementary information S6 for full description of method. Immediately prior to the boosting injection, photographs were taken of the forelimb and nuptial pad (Nikon D70, objective AF micro Nikkon 60 mm 1:2:8D). Photographs were analysed with ImageJ (National Institute of Health, Bethesda, MD - USA) to determine forelimb width and length and Adobe Photoshop CS6 for nuptial pad size and colour (see Supplementary Figures S1 and S2). Following breeding, nuptial pads were processed for analysis of histomorphology (see above). Photographs were randomised and all analyses were undertaken with no knowledge of treatment.

### Statistics

Water quality data (temperature, dissolved oxygen, pH, unionised ammonia), mortality rate, morphometric data (weight, snout-vent length, GSI [weight of testis or ovary/bodyweight], oviduct weight, oocyte stages [pre-vitellogenic, vitellogenic, post-vitellogenic, atretic], testicular cell types [spermatogonia, spermatocytes, spermatozoa] and gene expression data [brains from stage 66 metamorphs]) were compared between groups using ANOVA. Post-hoc significance was assessed through the Holm-Šídák test (Kruskal-Wallis test with Dunn’s post-hoc-test was used for data that were not normally distributed). Hindlimb length was analysed using ANCOVA, with snout-vent length as the covariate. Sex ratio of stage 51–53, stage 55–58, metamorph and adults (data for post-metamorphic individuals was combined prior to analysis) were analysed using a chi^2^ test. Gonadal maturation (measured based on the most advanced sex cell type observed) in male/female metamorphs was compared using Fisher’s exact test. In stage 51–53 and 55–58 tadpoles, a putative sex (male/female) was assigned to individuals based on an unsupervised hierarchical/kmeans cluster analysis of gonadal mRNA level (*dmrt1*, *amh*, *foxl2*, *cyp19*). After splitting the data based on putative sex, the expression of *ar, dmrt1, cyp17, cyp19, amh* and *foxl2* were compared across treatment groups using MANCOVA with developmental stage as a covariate. Where significant differences occurred in the MANCOVA, this was followed with pairwise comparisons of individual genes between control and treatment groups (ANCOVA followed by Holm-Šídák test for significance). Ontogenetic change in gene expression was analysed using linear regression and relationships between genes were analysed using Pearsons’ (normally distributed) or Spearmans’ (not-normally distributed) within each group. Differences in regression slopes between control versus treatment groups was analysed using ANCOVA with developmental stage as a covariate. Data for *dmrt1* and *cyp17* were Log10 transformed prior to analysis in order to normalise the data. Normal linear modelling (LM) was used to analyse output from the breeding trials. For males, data from breeding trials 1 and 2 were combined (i.e. considered as repeated measures) and linear mixed effect models were used to confirm there were no correlations within each individual frog for any of the variables tested. LM was then used to investigate effects of treatment on time in amplexus, fertility (percentage fertilized eggs [square root transformation was used to normalise fertility data]), nuptial pad size, nuptial pad colour, number breeding glands, number keratinised hooks, forelimb width index, testosterone/corticosterone levels and mRNA levels of *ar*/*gr* in arm/brain/gonad. For all models, the time between the two breedings (‘interval’) and weight were also always included to control confounding influence of recovery time and/or size on reproductive endpoints. For analysis of amplexus and fertility, data were only included where only one individual frog of the competing pair was in amplexus after the initiation of egg laying. In addition, since characteristics of unexposed females had the potential to contribute to fertility, fecundity (number of eggs laid) and female weight were also included in both models. The *p* value threshold below which effects were deemed significant was 0.05.

### Ethics

All experiments were approved by the University of Uppsala and carried out in accordance with relevant guidelines and regulations.

## Results

### Exposure Conditions

Water quality parameters in the different treatment groups were within the range for normal tadpole development and did not differ between groups (ANOVA; range: temperature, 25.4–27.1 °C; dissolved oxygen, 20.9–99.3%; pH, 7.19–8.25; unionised ammonia, 0–736 μg/L). Water conductivity ranged from 448–586 μS). Measured linuron and flutamide concentrations in dosed tanks were between 75% and 90% of nominal after water changes and did not differ between tanks within each treatment group (see supplementary Table S2). Neither linuron (LOD = 0.02 µg/L) nor flutamide (LOD = 5 µg/L) were detected in control tanks.

### Survival, Development and Gross Gonadal Morphology

Mortality rate (Supplementary Fig. S3), weight/length at metamorphosis or time to metamorphosis (Supplementary Fig. S4) did not differ between treatments but the hindlimb length at metamorphosis was greater in all exposed groups (males and females combined) compared with control (ANCOVA, post-hoc *p* < 0.001, Supplementary Fig. S4). In adults, there were no differences between treatment groups for total body weight, oviduct weight or GSI, except for females in response to the linuron high treatment where post-breeding the body weight was elevated (ANOVA, Holm-Sidak *p* = 0.02, Supplementary Fig. S5 and S6).

### Gonadal Development

**Figure 2:**
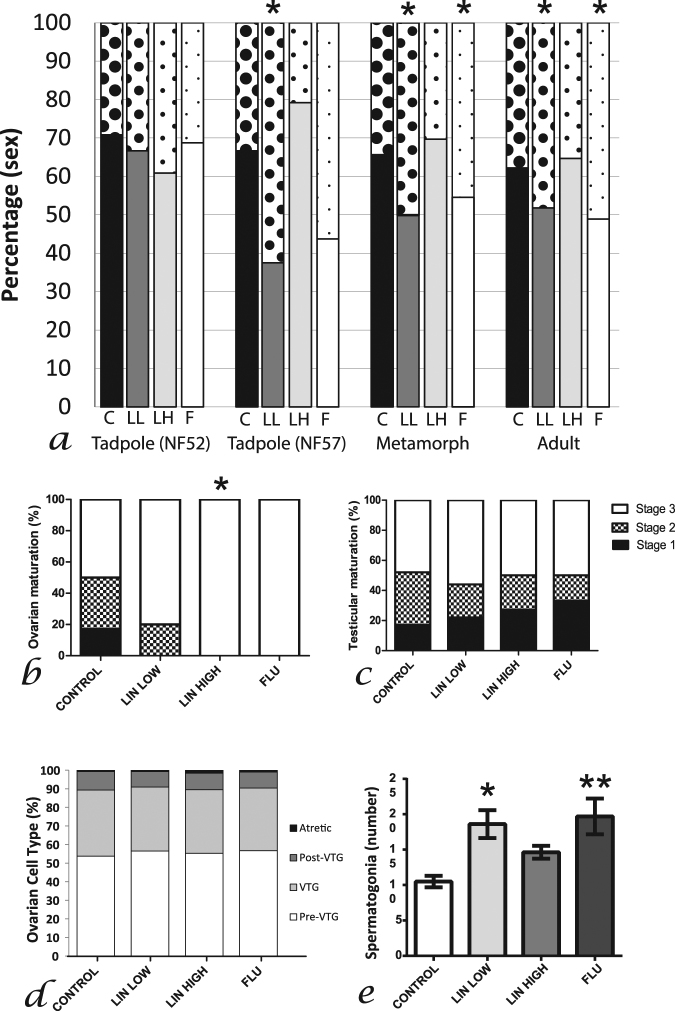
Sex ratio (**a**) in stage 51–53 tadpoles (control/linuron low *n* = 24, linuron high *n* = 23, flutamide *n* = 16), stage 55–58 tadpoles (control/linuron high *n* = 24, linuron low/flutamide *n* = 16), metamorphs (control *n* = 37, linuron low *n* = 24, linuron high *n* = 33, flutamide *n* = 11) and adults (control *n* = 82, linuron low *n* = 84, linuron high *n* = 85, flutamide *n* = 47). Solid bars = males, dotted bars = females. Gonadal differentiation in female (**b**) control *n* = 12, linuron low *n* = 10, linuron high *n* = 9, flutamide *n* = 4) and male (**c**) control *n* = 23, linuron low *n* = 10, linuron high *n* = 22, flutamide *n* = 5) metamorphs, and female (D: control/linuron low/linuron high *n* = 9, flutamide *n* = 7) and male (E: control/linuron low/linuron high/ flutamide: *n* = 10) adults. ^*^indicates significant difference compared with control group.

Sex ratio did not differ between treatments during the period of sex determination (stage ‘52’ (51–53)), (chi^2^, Fig. 2a), however differences were observed during sex differentiation (stage ‘57’ (55–58), chi^2^, *p* = 0.02, Fig. 2a): more females were found in the linuron low treatment group (62%: chi^2^, *p* = 0.03) compared to controls (32% females). This effect was also observed in post-metamorphic individuals in linuron low (chi^2^, *p* = 0.03, Fig. 2a) and flutamide (chi^2^, *p* = 0.04, Fig. 2a) treatment groups. Treatment related effects on gonadal maturation were observed in metamorphs and adults. In metamorphs, diplotenic oocytes were observed in all females in response to linuron high treatment, compared with 50% of control females (chi^2^, *p* = 0.04, Fig. 2b). No differences were observed in testicular maturation. In adult females, no treatment-related impact on ovarian maturity was observed (Fig. 2d) whereas in adult males, the number of spermatogonia (per seminiferous tubule) was higher in the linuron low and flutamide treatment groups compared with controls (ANOVA, post-hoc *p* < 0.01, Fig. 2e). In both treatment groups the mean numbers of spermatogonia per seminiferous tubule were approximately twice that of the controls (19/20, compared with 10). The diameter of the seminiferous tubule was larger in linuron high treated individuals compared to controls (Dunn’s, *p* < 0.01: median (25% percentile): control – 0.16(0.14); linuron low – 0.175(0.168); linuron high – 0.19(0.17); flutamide – 0.19(0.17)). No other differences between treatment groups were observed (see Supplementary Fig. S7 and S8 for photomicrographs showing features analysed in metamorph/adult gonads and Supplementary Tables S3 and S4 for full results for adult testis histopathology).

### Sex Related Gene Expression

**Figure 3:**
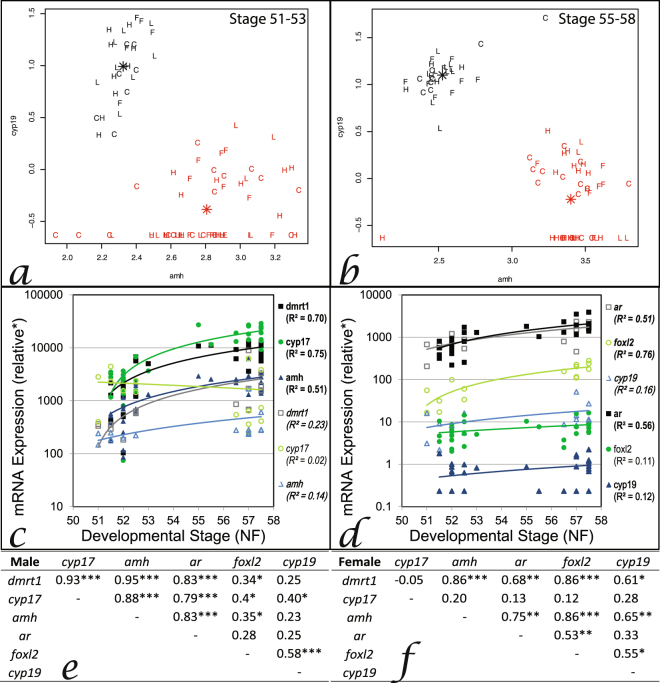
Relationship between *cyp19* and *amh* for distinguishing gonadal sex in tadpole gonado-mesonephros complex (GMC: a,b), gonadal ontogeny of gene expression (*dmrt1, cyp17, amh, ar, foxl2, cyp19*) in control tadpoles (**c**,**d**) and the interrelationships between these genes in control tadpole GMC (**e**,**f**). Unsupervised cluster analysis was used to delineate ‘males’ (black) and ‘females’ (red) during sex determination (**a**) and sex differentiation (**b**) for all treatment groups collectively (data not shown for *dmrt1 v. foxl2/cyp19* or *amh v. foxl2* as outcomes of the analysis did not differ). Each letter represents one individual and the asterices indicate the centre of each cluster (C=control, L=linuron low, H=linuron high, F=flutamide). Ontogeny of gene expression in males (normal, filled symbols) and females (italic, open symbols) tadpole GMC (**c**,**d**), bold indicates significant change during ontogeny using linear regression analysis ^*^expression is relative to gonad pool standards (see methods for details). Interrelationships between gene expression in male (**e**) and female (**f**) GMC analysed using Pearsons’ (or Spearmans’ for non-parametric data), ^*^p < 0.05, ^**^p < 0.01, ^***^p < 0.001.

Across all groups (controls and treated) and tadpole developmental stages (sex determination [stage 51−53]/differentiation [stage 55−58]), individuals could be separated into two groups based on mRNA levels of *dmrt1*, *amh*, *foxl2* and *cyp19* in the GMC. Combinations of levels of either *dmrt1* or *amh* plotted against either *foxl2* or *cyp19* level consistently separated animals into two groups (unsupervised cluster analysis, Fig. 3a,b). Based on the knowledge of the expression patterns for these genes that differs in males *versus* females during gonadal development (see ‘introduction’), putative sex of tadpoles was assigned. *Cyp17* mRNA levels were sexually dimorphic at sex differentiation only. *Ar* expression did not show any sexual dimorphism at either sex determination or differentiation (Fig. 3c,d). In both males and females, the mRNA levels of *dmrt1*, *ar*, *amh* and *cyp19* increased during tadpole ontogeny encompassing both sex determination and differentiation (stages 51–58: *p* < 0.05; R^2^ > 0.12, Fig. 3c,d). During ontogeny, mRNA levels of *cyp17* increased in males only (R^2^ > 0.75; Fig. 3c), whereas the expression of *foxl2* increased in females only (R^2^ > 0.76; Fig. 3d). Sex-specific relationships (positive correlations) were seen for the expression of some of these genes: In males, *cyp17* was positively correlated with *dmrt1*/*amh*/*ar*/*foxl2*/*cyp19* (*p* < 0.05; R^2^ > 0.40; Fig. 3e); In females, *cyp19* was positively correlated with *dmrt1*/*amh*; and *foxl2* was correlated with *ar* (*p* < 0.05; R^2^ > 0.53; Fig. 3f). These findings in the gonad contrasted with that in the brains of tadpoles where there were very few changes in expression during ontogeny nor were there any obvious correlations between individuals in the expressed genes (see Supplementary Fig. S10).

### Effects of Treatments on Gene Expression in Tadpoles and Metamorphs

**Figure 4:**
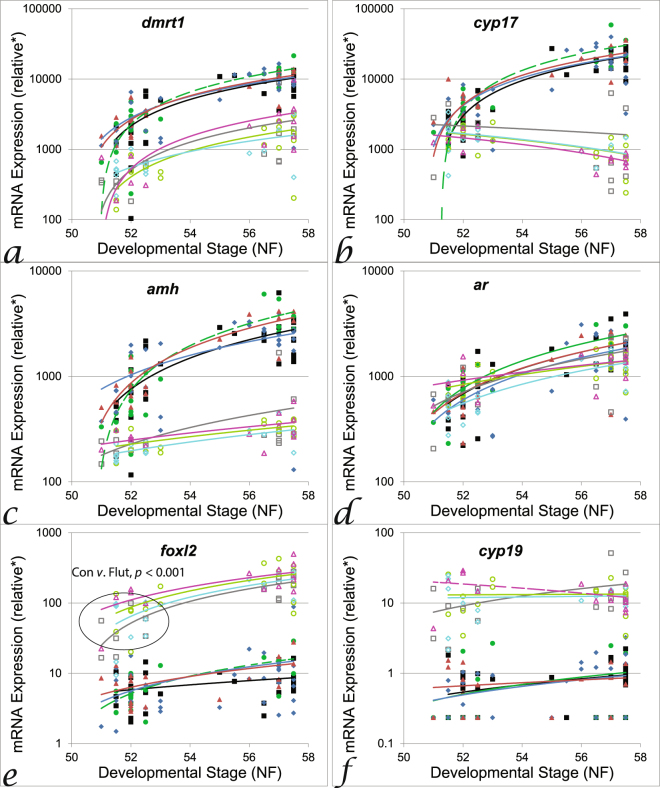
Effects of anti-androgens on expression of target genes during gonad ontogeny (NF stage 51–58). Linear regression of *dmrt1* (**a**), *cyp17* (**b**), *amh* (**c**), *ar* (**d**), *foxl2* (**e**) and *cyp19* (**f**). Black lines = control males (*dmrt1* r^2^ = 0.59, *cyp17* r^2^ = 0.62, *amh* r^2^ = 0.64, *foxl2/cyp19* r^2^ = ns) green lines = linuron low males (*dmrt1* r^2^ = 0.57, *cyp17* r^2^ = 0.67, *amh* r^2^ = 0.53, *foxl2/cyp19* r^2^ = ns), blue lines = linuron high males (*dmrt1* r^2^ = 0.79, *cyp17* r^2^ = 0.51, *amh* r^2^ = 0.65, *foxl2* r^2^ = 0.58, *cyp19* r^2^ = ns), red lines = flutamide males (*dmrt1* r^2^ = 0.81, *cyp17* r^2^ = 0.83, *amh* r^2^ = 0.9, *foxl2* r^2^ = 0.33, *cyp19* r^2^ = ns), grey lines = control females (*dmrt1* r^2^ = 0.41, *cyp17* r^2^ = ns, *amh* r^2^ = 0.33, *foxl2* r^2^ = 0.52, *cyp19* r^2^ = ns), light green = linuron low females (*dmrt1* r^2^ = 0.37, *cyp17* r^2^ = ns, *amh* r^2^ = 0.79, *foxl2* r^2^ = 0.81, *cyp19* r^2^ = ns), light blue = linuron high females (*dmrt1* r^2^ = 0.42, *cyp17* r^2^ = ns, *amh* r^2^ = ns, *foxl2* r^2^ = 0.31, *cyp19* r^2^ = 0.35), pink = flutamide females (*dmrt1* r^2^ = ns, *cyp17* r^2^ = ns, *amh* r^2^ = 0.32, *foxl2* r^2^ = 0.50, *cyp19* r^2^ = ns); with matching filled (male) or open (female) symbols. For MANCOVA analysis during sex determination (stage 51–53) or sex differentiation (stage 55–58) effects of flutamide were observed on *foxl2* levels in ‘females’ (see Supplementary Table S5 for numerical values/more details). Dashed lines represent significant differences in the linear regression slope compared with controls (ANCOVA, stage as a covariate). *relative gene expression compared to gonad pool standards.

**Figure 5:**
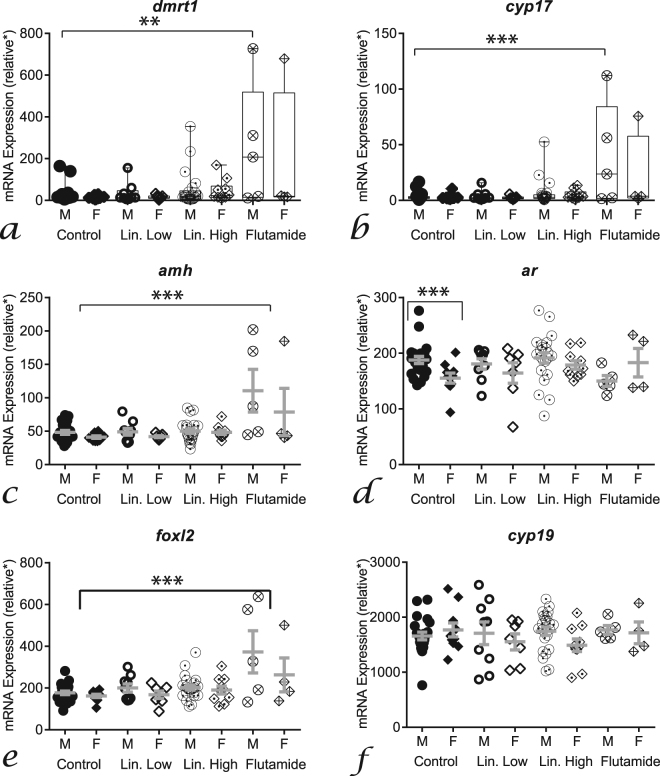
Effects of antiandrogens on expression of target genes in the brain in both male (circles) and female (diamonds) metamorphs (NF stage 66). *Dmrt1* (**a**), *cyp17* (**b**), *amh* (**c**), *ar* (**d**), *foxl2* (**e**) and *cyp19* (**f**) expression in controls (filled symbols), linuron low (open symbols), linuron high (dotted symbols) and flutamide (crossed symbols). Control male, *n* = 22; control female, *n* = 10; linuron low male, *n* = 9; linuron low female, *n* = 7; linuron high male, *n* = 22; linuron high female, *n* = 10; flutamide male, *n* = 5; flutamide female, *n* = 4. ^*^relative gene expression compared to brain pool standards (see methods for details).

Anti-androgen treatments affected mRNA levels in the GMC at sex determination (stage 51−53, Fig. 4 and Supplementary Table S5) and during ontogeny (stage 51–58; Fig. 4), but not at sex differentiation (stage 55−58). In females at sex determination, *foxl2* expression was elevated in response to flutamide treatment (MANCOVA *p* = 0.05, post-hoc *p* = 0.01, Fig. 4e and Supplementary Table S5). In males during ontogeny, there was a progressively higher level of expression of *dmrt1*, *cyp17* and *amh* (steeper slope) in response to linuron low treatment (ANCOVA *p* < 0.04, Fig. 4a–c). Effects of anti-androgen treatments in brains were only observed during ontogeny (Supplementary Table S6 for sex determination/differentiation data). In brains of males, there was a progressively lower level of expression of *ar* (R^2^ = 0.12) in the flutamide treatment compared with controls (R^2^ = 0.31; ANCOVA *p* < 0.05, Supplementary Fig. S11). In brains of females, there was a progressively greater level of expression of *foxl2* (R^2^ = 0.17) in the high linuron treatment group compared with controls (R^2^ = −0.02; ANCOVA *p* < 0.05, Supplementary Fig. S11). Differences in the interrelationships between the expression of the tested genes in treated groups compared to controls were observed in the GMC (see Supplementary Table 7 for details) but not in the brain (Supplementary Fig. 10). In metamorph brains, *ar* expression was sexually dimorphic in control organisms (*t*-test, *p* < 0.001), but this was not observed in treatment groups (Fig. 5). There was also a higher mRNA level of *dmrt1* (x14), *cyp17* (x9), *amh* (x2.3) and *foxl2* (x2) in the brains of the flutamide treatment group compared with controls (ANOVA, post-hoc *p* < 0.05, Fig. 5).

### Breeding Behaviour and Fertility in Adults

There was little variability in the time taken for frogs to enter into amplexus (at the second observation: 105 minutes (60 + 45 minutes)) or in the timing of egg laying (at the fourth observation: 195 minutes (60 + 45 + 45 + 45)) within the confines of the observation frequency. Females did not spawn in 7 cases (total=148 trials) and these data were excluded. Out of the remaining 141 male trials, in 79% (111) of cases, only one male was observed in amplexus after the female had started spawning and this ‘winning’ male was attributed the observed fertility and time in amplexus. Thus, data for the remaining 21% (30) of the trials, where it was unclear which male was responsible for the fertility/time in amplexus, were excluded. Amplectant frogs naturally separated in 82% of cases (6 hours after initiation of amplexus). There were no effects of treatments on time in amplexus, however, fertility was reduced in response to linuron low treatment (LM, *p* = 0.018, Table 1). Female factors were positively correlated with both fertility (egg number/female weight, LM *p* < 0.01) and time in amplexus (LM, < 0.01, Table 1).

**Table 1:** Effects of treatment on fertility and amplexus in male frogs (linear modelling).

Fertility	Mean(SE)	Estimate	Error	*P* value
Control	46.4(5.2)	−12.9	17.1	—
Linuron Low	37.5(4.4)	−12.1	5.07	0.018
Linuron High	46.8(3.8)	−2.12	4.85	0.66
Flutamide	46.4(5.2)	0.47	6.12	0.94
Interval	*factor*	0.07	0.15	0.62
Male Weight	*factor*	−0.02	1.5	0.98
Female Weight	*factor*	2.22	0.82	0.007
Egg Number	*factor*	0.39	0.12	0.002
*Model*				*4.1E-4*
**Amplexus**	**Mean(SE)**	**Estimate**	**Error**	***P*** **value**
Control	246(14.0)	−4.01	1.29	—
Linuron Low	253(15.0)	−0.07	0.31	0.81
Linuron High	282(10.3)	0.43	0.28	0.13
Flutamide	243(19.7)	0.20	0.37	0.59
Interval	*factor*	−0.04	0.009	<0.001
Male Weight	*factor*	0.08	0.08	0.35
Egg number	*factor*	0.03	0.007	<0.001
Female Weight	*factor*	−0.06	0.05	0.35
*Model*				*5.5E-5*

### Secondary Sexual Characteristics, Plasma Hormone levels and Gene Expression in Adults

Nuptial pad size and forelimb width index were positively correlated with male body weight (LM, *p* = 0.019). In response to linuron low treatment, there was a decrease in the size of the nuptial pad, the number of breeding glands and the number of hooks (LM, *p* < 0.01, Table 2). In contrast, linuron high treatment resulted in the development of a larger nuptial pad (LM, *p* = 0.035, Table 2) with a darker colour (LM, *p* = 0.008, Table 2) compared to controls. Similarly, in the flutamide treatment group, the nuptial pad was larger and had a higher number of hooks compared with controls (LM, *p* < 0.001)). Forelimb width index was reduced in response to all treatments (LM, *p* < 0.01, Table 2). Higher mRNA levels of *ar* were observed in the forelimb in response to flutamide treatments (LM, *p* = 0.02, Table 3) and higher mRNA levels of *gr* were observed in the brain of linuron high treated individuals (LM, *p* = 0.03, Table 3). No other effects were observed on mRNA levels (see Supplementary table S8). Testosterone levels were higher in all treatment groups compared with controls (LM, *p* < 0.01, Table 3). No effects on corticosterone were observed (Table 3).

**Table 2:** Effects of treatment on secondary sex characters in male frogs.

Nuptial Size	Mean(SE)	Estimate	Error	*P* value
Control	9.5(0.3)	6.72	1.30	—
Linuron Low	8.4(0.3)	−1.06	0.47	**0.024**
Linuron High	10.5(0.4)	0.96	0.45	**0.035**
Flutamide	11.6(0.7)	2.27	0.61	**<0.001**
Male Weight	*factor*	0.32	0.14	**0.019**
Interval	*factor*	−0.01	0.01	0.24
*Model*				*2.0E-07*
**Nuptial Colour**	**Mean(SE)**	**Estimate**	**Error**	***P*** **value**
Control	109.6(3.4)	101.2	12.6	*—*
Linuron Low	104.0(3.1)	−5.8	4.5	0.20
Linuron High	99.1(3.2)	−11.7	4.4	**0.008**
Flutamide	101.9(5.5)	−6.59	5.9	0.26
Male Weight	*factor*	−0.06	1.31	0.96
Interval	*factor*	−0.29	0.13	**0.03**
*Model*				*4.1E-02*
**No**. **Glands**	**Mean(SE)**	**Estimate**	**Error**	***P*** **value**
Control	25.8(1.28)	27.9	9.48	—
Linuron Low	15.5(1.16)	−10.4	3.11	**0.001**
Linuron High	24.0(1.36)	−1.57	3.07	0.61
Flutamide	28.9(1.87)	2.83	3.71	0.44
Weight	*factor*	−0.38	0.90	0.67
Interval	*factor*	0.05	0.15	0.73
*Model*				
**No**. **Hooks**	**Mean(SE)**	**Estimate**	**Error**	***P*** **value**
Control	115(8.0)	139	61.0	—
Linuron Low	74.2(6.4)	−43.6	20.0	**0.03**
Linuron High	140(9.6)	22.5	19.8	0.26
Flutamide	187(13)	78.4	23.9	**0.001**
Male Weight	*factor*	1.05	5.78	0.86
Interval	*factor*	−1.06	0.00	0.20
*Model*				*4.4E-4*
**Forelimb Width**	**Mean(SE)**	**Estimate**	**Error**	**P value**
Control	0.72(0.01)	0.57	0.03	—
Linuron Low	0.69(0.01)	−0.03	0.01	**0.01**
Linuron High	0.69(0.01)	−0.03	0.01	**0.002**
Flutamide	0.67(0.01)	−0.04	0.01	**0.005**
Male Weight	*factor*	0.02	0.003	**2.0E-07**
Interval	*factor*	0.0009	0.0003	**0.007**
*Model*				*2.4E-09*

**Table 3:** Effects of treatment on hormone levels and mRNA levels in male frogs.

Testosterone	Mean(SE)	Estimate	Error	*P* value
Control	9.5(0.7)	−3.80	4.39	—
Linuron Low	12.3(0.7)	3.24	1.46	**0.028**
Linuron High	12.9(0.5)	3.20	1.43	**0.026**
Flutamide	14.2(1.3)	4.38	1.84	**0.019**
Male Weight	*factor*	1.25	0.42	**0.003**
Interval	*factor*	0.08	0.07	0.25
*Model*				*2.7E-03*
**Corticosterone**	**Mean(SE)**	**Estimate**	**Error**	***P*** **value**
Control	3.1(0.2)	14.7	3.52	*—*
Linuron Low	4.8(0.5)	1.17	1.16	0.32
Linuron High	5.2(0.9)	1.81	1.14	0.12
Flutamide	3.7(0.5)	1.30	1.48	0.38
Male Weight	*factor*	−0.79	0.34	**0.02**
Interval	*factor*	−0.17	0.06	**0.003**
*Model*				*4.1E-02*
**Arm** ***ar***	**Mean(SE)**	**Estimate**	**Error**	***P*** **value**
Control	0.91(0.01)	0.94	0.08	—
Linuron Low	0.86(0.01)	−0.04	0.03	0.1
Linuron High	0.86(0.01)	−0.04	0.03	0.2
Flutamide	1.00(0.01)	0.09	0.04	**0.02**
Weight	*factor*	0.002	0.008	0.75
Interval	*factor*	0.0005	0.002	0.76
*Model*				*1.0E-02*
**Brain** ***gr***	**Mean(SE)**	**Estimate**	**Error**	***P*** **value**
Control	0.87(0.01)	1.02	0.06	—
Linuron Low	0.89(0.01)	0.02	0.02	0.4
Linuron High	0.91(0.01)	0.04	0.02	**0.03**
Flutamide	0.87(0.01)	0.004	0.03	0.87
Weight	*factor*	−0.01	0.006	**0.03**
Interval	*factor*	−0.001	0.001	0.14
*Model*				*2.8E-02*

^*^Results for brain/testis *ar* and arm/testis *gr* are shown in Supplementary Table S8.

## Discussion

Anti-androgenic chemicals represent some of the most prevalent endocrine disrupting chemicals in the environment but there has been relatively little study on the effects of these chemicals in non-mammalian animals. These anti-androgenic types of pollutants are of particular relevance to frogs, which are likely to be exposed to them during early life stages (tadpoles) in the aquatic environment, and coinciding with the time of gonadal sex differentiation and development. In our experimental system the rate of tadpole, metamorph, and frog development, including gonadal development, were similar to those reported previously for lifecycle studies with *X. tropicalis*^37,46^. The skew in sex from a 50:50 male:female ratio in controls (at between 70–63% males, depending on life stage analysed) in this study has also been reported for other laboratory populations of *X. tropicalis*, albeit the ratio has been reported to vary more widely in some cases (males: 29%^37^: 60%^46^). We report effects of anti-androgenic treatments on gonadal development (sex ratio, development of germ cells), reproduction (spermatogenesis, male fertility), secondary sexual characteristics (nuptial pad, forelimb width) as well as on expression of, and interrelationships between, selected genes integral to sex determination/differentiation. Exposure to the lower concentration of linuron (9 µg/L) and flutamide (50 µg/L) feminised the sex ratio and resulted in an increased number of spermatogonia in the adult testis. In addition, the low (9 µg/L) linuron exposure resulted in reduced nuptial pad size and fertility in adult male frogs. Few effects were observed in response to the higher linuron concentration.

We first established that a selected set of genes could be used to distinguish sex during early life prior to when the gonad sex can be distinguished histologically. In agreement with previous studies in amphibians for equivalent developmental stages (see introduction), *dmrt1*, *amh*, *foxl2*, and *cyp19* showed sexual dimorphism in the GMC at sex determination (NF stage 51–53) and sex differentiation (NF stage 55–58). Although we were not able to confirm that the pattern of mRNA levels reflected actual gonadal sex the likelihood for this was very high given that the subsequent sex ratio in the control populations studied reflected the sex ratio outcome and the knowledge that the involvement of these genes in sex is highly conserved across vertebrates. We therefore used inter-relationships in mRNA levels of *dmrt1, amh, foxl2* and *cyp19* to assign putative sex. Animals with higher levels of *amh*/*dmrt1* were assigned as putative ‘males’ and those with higher levels of *cyp19/foxl2* assigned as putative ‘females’ (approximately 10-fold difference), with identical sex characterisation using any combination of ‘male’ genes *versus* ‘female’ genes. Assigning sex in early life stages of amphibians based on the levels of specific mRNAs has been adopted previously using *cyp19* in *X. tropicalis* and *L. sylvatica*^12^, and *cyp19* combined with *amh* in *X. tropicalis* (*cyp19* and *amh*^19,47^). In our study, exposure to the anti-androgen treatments did not alter the sex defining patterns of mRNA levels of ‘males’ and ‘females’. This may not always be the case, however, as some chemical treatments may be more potent than these in affecting these gene targets and thus interfere with this approach as a way to distinguish genetic sex. Once sex had been assigned, in accordance with the role these genes are known to play in sexual development in other animals (see introduction), we found that in assigned ‘males’ mRNA levels of *dmrt1*, *amh* and *cyp17* increased during ontogeny (encompassing sex determination and differentiation: NF stage 51–58), whereas in ‘females’ *foxl2* increased. Our findings support the role of *cyp17* in sexual differentiation (NF stage 55–58), as levels increased dramatically in ‘males’ (R^2^ = 0.75) and not in ‘females’ (R^2^ = −0.02) during ontogeny (NF stage 51–58). The *cyp17* enzyme converts progestogens to androgens and has previously been reported to be essential for normal testicular differentiation in wrinkled frogs^16^. In our study, *cyp17* was also positively correlated with all tested genes in ‘males’ but not with any of the tested genes in ‘females’, suggesting an integral role of *cyp17* in male sexual development, but not female development. Levels of *ar* mRNA were not sexually dimorphic in the GMC stages analysed (in agreement with previous reports^22,23^). *Ar*, however, may play an important role in sexual development as its transcript level increased during ontogeny in both sexes. *Ar* levels were also positively correlated with the mRNA levels of genes associated with masculinisation (*dmrt1* and *cyp17*) in ‘males’, and the mRNA levels of the feminising gene *foxl2* in ‘females’. Androgens have previously been shown to regulate expression of *dmrt1* and *cyp17* through *ar* during sexual differentiation in *R. rugosa*^21^. By contrast, in female snapping turtles (*Chelydra serpentina*) *ar* has been reported to be functionally linked *foxl2* during sexual differentiation^48^. To further investigate a possible link between *ar* and other genes important for sexual development, we searched for putative binding sites for the androgen response element in the upstream promoter region of target genes using the JASPAR online database of transcription factors (http://jaspar.genereg.net/). We found a similar interaction score (predicted DNA protein binding sites) of the human androgen response element with *X. tropicalis dmrt1* (11.9), *cyp17* (12.0), *foxl2* (12.4) and human *sox9* (12.9), which were higher than for the housekeeping gene *rpl8* (10.0) (Supplementary Table S10). In metamorphs, with the exception of *ar*, none of the genes tested showed sexually dimorphic expression in brain tissue at any stages of development. This supports a generalised assumption that the brain has a less important role than the gonad in driving sexual determination/differentiation of the gonad.

We found a feminised sex ratio in response to flutamide and the lower exposure concentration of linuron in tadpoles post-sexual differentiation (NF stage 55–58). For the linuron low treatment group, the feminised sex ratio persisted post-metamorphosis. Flutamide exposure has been shown to induce complete feminisation in *R. rugosa*^23^ but at a concentration higher than adopted in our studies on *Xenopus tropicalis* (50 μg/L *versus* 138 μg/L). These differences in the level of effect may also relate to possible differences in species sensitivity. In the flutamide treatment group there was a higher level of *foxl2* (2.4-fold) during sex determination (NF stage 51–53) compared with controls. There was a tendency for an increased mRNA level of *foxl2* also in response to the linuron low treatment (2-fold, not statistically significant, *p* = 0.06). Increased levels of *foxl2* during sexual determination are consistent with the feminisation of sex ratio observed. Similar effects were reported in snapping turtle (*Chelydra serpentina*) embryos exposed to dihydrotestosterone (a feminising agent in this species) where at a temperature that normally produces a mixed sex outcome where an almost complete feminisation of the population resulted (98% *versus* 66% females in controls) and this was associated with a 2-fold higher level of *foxl2*. Furthermore, these effects were reversed upon simultaneous exposure with flutamide^48^. In brown trout (*Salmo trutta*), treatment with androgens at levels that cause masculinisation are also associated with lower level of *foxl2*^49^. The feminised sex ratio observed in response to linuron low and flutamide in our study may thus have been mediated through a disruption *ar*/*foxl2* signalling during sexual development, as has been suggested to occur for chemically induced feminisation of snapping turtles^48^.

In the breeding trials, there was a high success rate in the mating groups (>95%) and both the time in amplexus and the number of eggs laid in controls were comparable to those reported previously in *X. tropicalis*^37^. In our investigations into the relationships between the physical features of *X. tropicalis* and breeding, we show nuptial pad size was positively correlated with body weight. This contrasts to that reported for wild *Bufo bufo* where no correlation was observed^50^, and may reflect differences in the role of the nuptial pad for species with different breeding patterns; *B. bufo* employs an explosive breeding mode^51^ whereas the breeding mode for *X. tropicalis* is ‘prolonged’^52^ (elaborate courtship rituals employed by few competing males compared to ‘explosive’ breeders, where males scramble to mate with the females in ‘breeding balls’). We also show that the number of eggs laid (fecundity) by female *X. tropicalis* was positively correlated with the proportion of eggs fertilised, indicating that more fecund females may have better overall egg quality (a greater proportion of eggs that are fertile). A similar relationship between fertility and fecundity has been reported in fish^53^. It may also be the case, however, that higher fecundity has a positive impact on gamete production by the male and more fecund females stimulate males more effectively, thereby driving fertilisation success. Although these findings are perhaps not surprising, they have not often been reported in the scientific literature.

Post-breeding, in the adult males, the early life linuron low and flutamide treatments resulted in an increase in the amount of spermatogonia. No comparable early life exposure studies are known in amphibians, however, androgen treatment of adult life stages has been shown to result in a reduction in the amount of undifferentiated spermatogonia in the testis^54^. Flutamide exposure in adult Murray rainbow fish (*Melanotaenia fluviatilis*), albeit at much higher exposure levels (500 or 1000 µg/L), resulted in higher numbers of spermatogonia^55^. In response to the linuron low treatment, this seemingly “anti-androgenic” effect on spermatogenesis was accompanied by reduced fertility. No such effect on fertility was observed in the flutamide group. It is not known why linuron and flutamide treatments had different effects on fertility, however, we need to be careful not to assume that all anti-androgenic chemicals work in exactly the same way. Indeed, although linuron and flutamide are androgen receptor antagonists^27^, linuron has also been shown to alter testicular steroidogenesis *in vitro*^56^. We did observe that mRNA levels of genes known to be important for male sexual differentiation were dramatically increased in the brains of metamorphs for the flutamide treatment group compared to controls (*dmrt1* [x14] & *cyp17* [x9]), but this was not the case in response to the low linuron exposure group. It is plausible that masculinisation of the brain in flutamide treated individuals partially ameliorated long-term effects of the anti-androgenic treatment but this is a hypothesis only. Long-term effects on gene transcript levels in the brain have previously been reported in *X. tropicalis* exposed to ethinylestradiol during early life, whereby brain mRNA levels of *er-alpha* were reduced in the resulting adults^38^. In addition to reduced fertility, individuals from the linuron low group also had a smaller nuptial pad, containing fewer hooks and fewer breeding glands compared to controls. This is in agreement with the known interplay between reproductive condition and nuptial pad size/colour, whereby reproductively active male frogs have large and dark nuptial pads^29^. Somewhat surprisingly, individuals from the flutamide exposure group had larger nuptial pads containing more hooks compared to controls. It is unknown why these differences occurred, however, the mRNA level of *ar* was higher in the forelimbs of flutamide treated frogs compared to the other groups, and this may have sensitised the tissue to circulating androgens.

In response to all anti-androgen treatments, androgen levels were higher in treated animals compared to the controls. There are no comparable studies on amphibians for effects of early life exposure to androgens or anti-androgens on hormone levels in adults, however, peripubertal exposure to the anti-androgenic fungicide vinclozolin in rats has been shown to increase testosterone levels^57^. Adult male frogs from the treated groups also had a smaller forelimb width compared with controls. This might be expected for an anti-androgenic effect as forelimb muscles are under the positive control of androgens in amphibians^58^. Therefore, although both higher androgen levels and smaller forelimb width may therefore be indicative of anti-androgenic effects, they are also somewhat contradictory effects since androgens are positively associated with forelimb muscle size. The reason for this disparity could be due to a mismatch between time of growth/development of forelimbs (juveniles/adults) and the time when the plasma samples were taken for analysis of androgen levels (adults post-breeding).

For the linuron low treatment there were effects consistent with effects of developmental exposure to an anti-androgen (i.e. effects on sex ratio, male germ cells, gene expression, secondary sex characters, fertility). However, this was not the case for the higher linuron exposure concentration (i.e. responses did not follow a monotonic dose-response relationship). Exposure to the higher concentration of linuron resulted in an accelerated rate of ovarian development, that has been reported previously also in juvenile catfish (*Clarius batrachus*) exposed to flutamide or endosulfan (an anti-androgenic pesticide^59^). We have no definitive explanation for this, however, the phenomena of so-called ‘non-monotonic’ dose response effects (or “hormesis”) – where a lower concentration can have a more marked effect than for a higher exposure concentration for a given endpoint is not an uncommon observation in studies on the effects of pollutants on the endocrine system of vertebrates^60^, including amphibians. Examples of this include for the feminisation of sex ratio in *X. laevis* in response to bisphenol A^61^. Proposed mechanisms for non-monotonic effects in response to EDCs include receptor down-regulation and desensitization at higher chemical exposure concentrations and organism response to treatments by alterations in the central hypothalamo-pituitary-gonadal axis mediated by endocrine negative feedback loops^60^. Our finding that linuron can affect fertility in male frogs at an exposure level reported for some aquatic environments (9 µg/L:^e.g.^^35,36^) highlights concern for the presence of this anti-androgen in freshwaters and supports the need for further work on the impacts of anti-androgens, and other endocrine disrupting chemicals, on reproduction in wild amphibian populations.

## Electronic supplementary material

Supplementary Information
