# Supplementary material for: Exposure to an anti-androgenic herbicide negatively impacts reproductive physiology and fertility in Xenopus tropicalis
Source: Sci Rep. 2018 Jun 14;8:9124. doi: 10.1038/s41598-018-27161-2 (PMC6002408; doi:10.1038/s41598-018-27161-2)
Supplement: Supplementary file 1 — Supplementary Information [file 41598_2018_27161_MOESM1_ESM.pdf]

Exposure to an anti-androgenic herbicide negatively impacts reproductive physiology and fertility in Xenopus tropicalis

Orton F*[a1], Säfholm M[a2], Janson E2, Carlsson Y[2], Eriksson A[2], Fick J[3], Uren Webster T[4], McMillan T[1], Leishman M[1], Verbruggen B[5], Economou T[5], Tyler CR[b5] and Berg C[b2].

1. University of the West of Scotland, UK; 2. Department of Environmental Toxicology, Uppsala University, Sweden; 3. Department of Chemistry, Umeå University, Sweden; 4. Swansea University, UK; 5. College of Life and Environmental Sciences, University of Exeter, UK.

aFirst Authors

bSenior authors

* corresponding author: frances.orton@uws.ac.uk

Supplementary methods S1 – Exposure conditions

Tadpoles were exposed to nominal concentrations of linuron ('low' - 32 nM/9 µg/L or 'high' – 181 nM/45 µg/L) or flutamide (181 nM/50 µg/L: purity > 98 %, Sigma Aldrich, USA) in acetone (0.0008%) or to acetone only (0.0008%: hereafter referred to as 'controls') using a semi-static system (50% water change, x3 per week) to maintain good water quality[1]. Aquatic half life for flutamide has been reported to be 49 days[2], though equivalent information for flutamide was not available, chemistry was undertaken to verify exposure concentrations (see table S2). Triplicate tanks were used for each experimental group (total = 12 tanks) and the placing of tanks in the experimental room was randomised. Tanks were placed in water channels to maintain constant temperature, and this was checked daily (26 ± 1 °C), together with conductivity of water used for water changes (505 ± 20 µS/cm). The photoperiod cycle was 12:12 light:dark. Tank water temperature, dissolved oxygen, pH and nitrite/ammonia levels were measured weekly. Flutamide and linuron concentrations were analysed using GCMS before water changes and immediately after water changes. For flutamide, analysis of the tank water concentrations were done bi-weekly in flutamide and control tanks (5 before + 5 after, see:[3]) and for linuron, analysis of tank water concentrations were done every three weeks in linuron and control tanks (3 before + 1 after, see:[4]). The chemical analyses were undertaken at Umea University (flutamide) and at the Swedish University of agricultural sciences (linuron).

1 Berg, C., Gyllenhammar, I. & Kvarnryd, M. Xenopus tropicalis as a Test System for Developmental and Reproductive Toxicity. *Journal of Toxicology and Environmental Health, Part A* **72**, 219-225, (2009).
2 U.S. EPA. Risks of Linuron Use to Federally Threatened California Red-legged Frog (Rana aurora draytonii). https://www3.epa.gov/pesticides/endanger/litstatus/effects/redleg-frog/linuron/determination.pdf. 2008
3 Grabic, R., Fick, J., Lindberg, R. H., Fedorova, G. & Tysklind, M. Multi-residue method for trace level determination of pharmaceuticals in environmental samples using liquid chromatography coupled to triple quadrupole mass spectrometry. *Talanta* **100**, 183-195, (2012).
4 Jansson, C. & Kreuger, J. Multiresidue Analysis of 95 Pesticides at Low Nanogram/Liter Levels in Surface Waters Using Online Preconcentration and High Performance Liquid Chromatography/Tandem Mass Spectrometry. *Journal of AOAC International* **93**, 1732-1747 (2010).

Supplementary methods S2 – Animal husbandry

Adult male and female *X.tropicalis* were obtained from Xenopus 1 (Dexter, USA) and housed in glass tanks in a flow-through system (12:12 light:dark cycle, 26 ± 1 °C, conductivity 505 ± 20 µS/cm) for approximately two years prior to this study. Four pairs of frogs were induced to mate using human chorionic gonadotropin (hCG) as previously described[4]. Thirty-eight tadpoles (18 hours post-fertilisation at 22^{0}C, stage 40) were selected from the 2 pairs of frogs with highest fertilisation rates (estimated, > 50%) and placed in experimental tanks (15 L, *n* = 76 tadpoles per tank). Tadpoles were initially fed Sera micron and fish flakes (Sera vipan baby, Sera, Heinsberg, Germany) until forelimbs could be observed, when their food was supplemented with Frog & Tadpole bites (HBH Pet Products, Springville USA). Metamorphs were fed Frog and Tadpole bites, gradually replaced by fish pellets during 1-3 month post-metamorphosis (Tropical Excel Color, Aquatic Nature, Roeselare, Belgium).

4 Pettersson, I., Arukwe, A., Lundstedt-Enkel, K., Mortensen, A. S. & Berg, C. Persistent sex-reversal and oviducal agenesis in adult Xenopus (Silurana) tropicalis frogs following larval exposure to the environmental pollutant ethynylestradiol. *Aquatic Toxicology* **79**, 356-365 (2006).

Supplementary methods S3 – Adult male histomorphology

For testis histomorphology, digital photos of a section cut through the centre of the right testis were captured with a photo-micrographic camera (Leica DFC 550, Leica AB, Kista). For each section analysed, a grid was overlaid and all seminiferous tubules that contained crossing gridlines (spacing: 0.7 mm) were selected for analysis (3-23 tubules, depending on the size of the testis). For each seminiferous tubule analysed, the number of germ cell nests (cyst-like structures within the luminal margins of the seminiferous tubule) and each nest was classified according to the most mature cell type observed: spermatocytes, spermatids or spermatozoa, using established criteria[5]. In addition, the number of spermatogonia per tubule and the number of spermatocytes in the largest spermatocyte nest in each tubule (2-20 nests per tubule) were counted. The amount of spermatozoa in the lumen of the tubule was assessed and assigned a score number (spermatozoa: 1 = tubules with little/no spermatozoa, 2 = tubules with spermatozoa in half of the lumen, 3 = tubules with lumen filled with spermatozoa)[6]. Testis and tubule diameter length and width (average calculated for data analysis) and the total number of seminiferous tubules were recorded. Testicular morphology was compared between treatments using mean values for each measured endpoint across tubules within individuals. Analysis was done directly or using ImageJ software (National Institute of Health, Bethesda, MD, USA) where appropriate. All slides were analysed without knowledge of exposure group.

5 Kalt, M. R. Morphology and kine ics of spermatogenesis in Xenopus laevis. *Journal of Experimental Zoology* **195**, 393-407, doi:10.1002/jez.1401950306 (1976).

6 Gyllenhammar, I., Holm, L., Eklund, R. & Berg, C. Reproductive toxicity in Xenopus tropicalis after developmental exposure to environmental concentrations of ethynylestradiol. Aquatic Toxicology 91, 171-178 (2009).

Table S1. Gene specific qPCR primers for *X.tropicalis.*

**Target gene**	**Primer (5' – 3')** sense	antisense	**Accession no.**	**Ta (ºC)**
amh[a]	GCTGCTGAAAAGAAGGATGC	AGTCCACCAACCATTCCAAA	-	55
foxl2	AGGGCAACTACAGGCGAAGA	GTGGGGACAGGTAGCCGTAG	ENSXETG00000008253[b]	60
cyp19a1	TGGGCCCCTCATTTCTTATG	ACCAGTGTTTCCTCGCCATT	NM_001097161.1	60
ar	ACCTGGTGTTTAATGAGTATCGC	CAGGAATAATGCTAAAGAGAAGGAG	XM_002941842.2	60
dmrt1	ACCATTTAGCAAGACTCGTAGC	TCCCTCCACATACAGAAGCG	XM_002935602.2	59.5
cyp17	GAGATAGCCTGCCAGACC	TTTCCAGACCTCCCTCAAC	NM_001127045.1	59.5
gr	CCATCTCACAGCATCAG	ACCTATTCCAGCCTTCT	-	60
rpl8[c]	CCCTCAACCATCAGGAGAGA	TCTTTGTACCACGCAGACGA	BC059744	62

[a]amh primers, Jansson E., Mattsson A., Goldstone J., Berg C., 2016

[b]ENSEMBL

[c]rpls8 primers, Langlois et al. 2010, Gen Comp Endocrin 417-427

Ta = annealing temperature.

Supplementary methods S4 – Molecular methods

For tadpoles and metamorphs, tissue samples (brain or GMC) were homogenized using a Bullet Blender® Storm 24 (speed 8, 1 min, 0.5 mm zirconium oxide bullets: Next Advance, Inc. New York USA) in sample lysis solu ion (30 µl). Total RNA was extracted using column based Aurum™ Total RNA Mini Kit (Bio-Rad Laboratories Inc. CA USA) according to the protocol. Isolated RNA was eluted with 40 µl elution solution and quantified with a NanoDrop (NanoDrop Technologies, Wilmington, DE, USA). Two samples from each RNA extraction run (18 samples) were also assessed for RNA integrity on 1.2 % agarose gel. cDNA was synthesised from total RNA in duplicate for each sample. Total RNA (300 ng) was reverse transcribed using iScript cDNA Synthesis Kit (Bio-Rad Laboratories Inc. CA USA) in an iCycler (5 minutes at 25°C, 30 minutes at 42°C, 5 minutes at 85°C, hold at 4°C: Bio-Rad Laboratories Inc. CA USA). cDNA product was diluted in water and stored at -20 °C un il further analysis. Negative reverse transcriptase controls (RTC), containing RNA template but without enzyme reverse transcriptase, were also included. For adults, RNA was extracted from the brains, gonads and arms using TRI reagent (Sigma-Aldrich) according to the manufacturer's instructions. RNA concentration and purity were assessed with a NanoDrop ND-1000 Spectrophotometer (NanoDrop Technologies, Wilmington, USA). cDNA was synthesised according to manufacturer's instructions from 2 µg of total RNA treated with RQ1 DNase (Promega, Southampton, UK) using random hexamers (MWG-Biotech) and M-MLV reverse transcriptase (Promega).

Primers for *foxl2* were designed using Primer3 [6] and were synthesised by Sigma-Aldrich (Haverhill, UK). All designed primer pairs were optimised for annealing temperature and the products were verified by sequencing at the Uppsala Genome Center (Uppsala, Sweden) and aligned with design primer pair products in ClustalW2 (European Bioinforma ics Ins itute, Cambridge, UK). The 20 µl qPCR reaction mixture consisted of 50 % iQ™ SYBR®Green Supermix (Bio-Rad Laboratories Inc. CA USA), 0.2 µM of forward and reverse primers and 0.05, 0.1, 0.5 or 1.0 ng/µl cDNA. The qPCR program included an enzyme activation step for 3 minutes at 95 °C and 40-45 cycles of 15 seconds at 95 °C and 30 seconds at primer specific annealing temperature. A melt curve analysis running from 55 °C to 95 °C generated a dissociation curve to confirm the presence of a single amplicon (Rotor-Gene 6000, Qiagen, Hilden, Germany). Primer sequences were available for ar:[7], *amh*[8], and cyp*19a1*[8]. Primers for *dmrt1, cyp17* and *gr* were designed with the Beacon Designer software (Premier Bios o f t International, Palo Alto, CA). All primers were purchased from MWG-Eurofin (Ebersburg, Germany) and verified using BLAST for alignment to the specific target genes. All designed primer pairs were optimised for annealing temperature. The expression of all gene targets were analysed with the CX96 ™ Real-Time System (C1000 ™ thermo cycler, Bio-Rad Laboratories, CA). The 15 µl qPCR reac ion mixture consisted of 50% iQ™ SYBR®Green Supermix (Bio-Rad Laboratories Inc. CA USA), 10 µM of forward and reverse primers and 0.001-35 ng cDNA. For *ar*, the qPCR program included an enzyme ac iva ion step for 3 minutes at 95°C and 45 cycles of 15 seconds at 95 °C/30 seconds at 60^0C. For *dmrt1* and *cyp17*, the qPCR program included an enzyme ac iva ion step for 5 minutes at 95 °C and 40 cycles of 10 seconds at 95 °C/30 seconds at 59.5^0C. Details on primer sequences and qPCR assay condi ions are shown in Supplementary, Table S1. Melt curve analyses were carried out for each target gene to confirm the presence of a single amplicon.

7 Koressaar, T. & Remm, M. Enhancements and modifica ions of primer design program Primer3. *Bioinformatics (Oxford, England)* **23**, 1289-1291 (2007).
8 Safholm, M., Jansson, E., Fick, J. & Berg, C. Mixture effects of levonorgestrel and ethinylestradiol: estrogenic biomarkers and hormone receptor mRNA expression during sexual programming. *Aquatic toxicology (Amsterdam, Netherlands)* **161**, 146-153 (2015).
9 Jansson, E., Mattsson, A., Goldstone, J. & Berg, C. Sex-dependent expression of an i-Müllerian hormone (amh) and amh receptor 2 during sex organ differen ia ion and characteriza ion of the Müllerian duct development in Xenopus tropicalis. *General and Comparative Endocrinology* **229**, 132-144 (2016).

Supplementary methods S5 – Breeding trials

Breeding of male X. tropicalis took place 6 months post-metamorphosis. During the week prior to breeding, males were weighed and individually marked within toe webbings (Visible Implant Elastomer Tags, Northwest Marine Technology, USA). A competitive breeding system was used, whereby 2 experimental males were placed with 1 unexposed female. Unexposed female frogs were of a similar age (~1.5 years) and size (~20 grams) and were obtained from Xenopus 1 (Dexter, USA) 2 weeks prior to the beginning of the breeding trials. Two breeding trials were undertaken with each experimental male, ensuring a similar recovery time between breeding trials (minimum of 10 days[10]). In total 158 trials were undertaken (92 trials for the first breeding, and 66 for the second breeding). One day prior to the breeding trial, the selected males (4 or 5 sets of two males from each treatment) and females (4 or 5 individuals) were placed in holding tanks (each set of two males in 6L tanks; all female frogs in a 15L tank) in the breeding room in water at a lower temperature (24 ± 1 °C) to stimulate breeding[1]. To induce breeding, male and female frogs were given two injections of hCG: priming (20 IU) and boosting (23 hours later, 100 IU). Immediately prior to the priming injection, male frogs were weighed, and immediately prior to the boosting injection, photographs were taken of the forelimb and nuptial pad (Nikon D70, objective AF micro Nikkon 60 mm 1:2:8D). Photographs were analysed with ImageJ (National Institute of Health, Bethesda, MD - USA) to determine forelimb width and length and Adobe Photoshop CS6 for nuptial pad size and colour (see Supplementary Figures S1 & S2). Photographs were randomised and all analyses were undertaken with no knowledge of treatment.

Following the hCG boosting injection, each set of two males was placed in a breeding tank (20L, darkened with plastic covering, containing 3 glass petri dishes), and a pre-weighed female was introduced. To minimise confounding effects, the boosting injection of the first or second male from each pair was alternated. Male frogs were given 20-25 minutes recovery time prior to addition of the female, and the order in which females were added to breeding tanks was randomised. The breeding trial for each tank was initiated when the female was placed in the breeding tank with the male pair. After 60 minutes, and thereafter every 45 minutes, tanks were assessed to identify the individual male in amplexus and whether spawning had occurred. Six hours after the first amplexus was observed – or if the frogs were not observed in amplexus for two of the consecutive measured time points – all frogs were removed from the breeding tanks (this always occurred within 10 hours of initiation of breeding trial). Photographs were taken of the tanks containing spawn immediately following removal of the frogs to determine the number of eggs spawned. A sub sample of the eggs spawned was then removed from each fertilisation tank and placed into glass petri dishes (2L, 26.50C in water channel). Fertilisation rate was assessed by comparing photographs taken immediately after their collection from the breeding tanks and those taken 26 hours after initiation of amplexus, when the hatched fertilised eggs could be distinguished by early embryo development (elongation of the spherical egg). See Supplemental methods S6 below for details on method optimisation. Immediately following the second breeding, male frogs were anaesthetised and sacrificed by pithing. Their snout-vent length was measured, and weights of the body and left testis weight were measured for calculation of gonado-somatic index (GSI). The right testis was fixed in NBF (4%) for histological analysis.

10. Harland. *Xenopus tropicalis* husbandry, Harland lab. https://tropicalis.berkeley.edu/ [accessed Dec 2014]

Supplementary methods S6 – fertility determination

Two factors were taken into account during optimisation:

1. Practicality: Development over 20 hours after amplexus had ceased (~26 hours after initiation of amplexus) allowed time to remove the fertilized eggs and get the tanks ready for the next batch of eggs for the following breeding trials (depending on amplexus behaviour, there could be as little as 30 minutes between the two).
2. Optimal timing: As embryo development starts directly at fertilization, 20 hours after amplexus ceased fertilized oocytesd should have reached at least Nieuwkoop and Faber 25 to 33 (depending on temperature) and fertilized eggs should be obvious/easy to identify.

Fertilization success was measured at 18, 19, 20 and 21 hours after amplexus ceased. In aquarium 1 (Figure 1) and aquarium 2 (Figure 2) 20 hours was shown to be sufficient to capture the vast majority of the fertilization that had occurred.

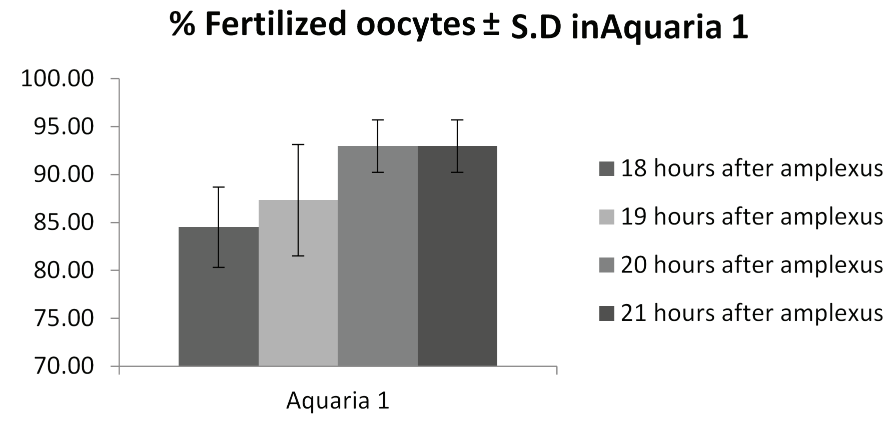

Figure 1. Percentage fertilization success in aquarium 1. Three separate petri dishes were cultivated over night in water bath and analyzed 18, 19, 20 and 21 hours after amplexus ceased. Data presented as mean ± S.D.

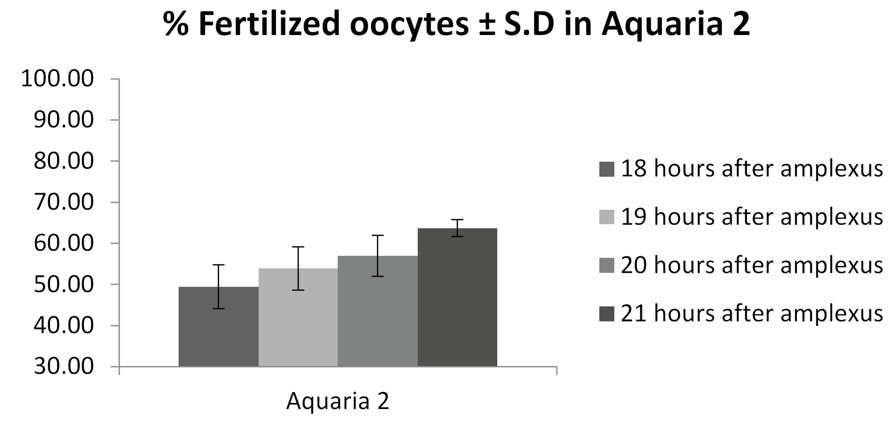

Figure 2. Percentage fertilization success in aquarium 2. Three separate petri dishes was cultivated over night in water bath and analyzed 18, 19, 20 and 21 hours after amplexus ceased. Data presented as mean ± S.D.

Figure S1. Measurement of forelimb width and length.

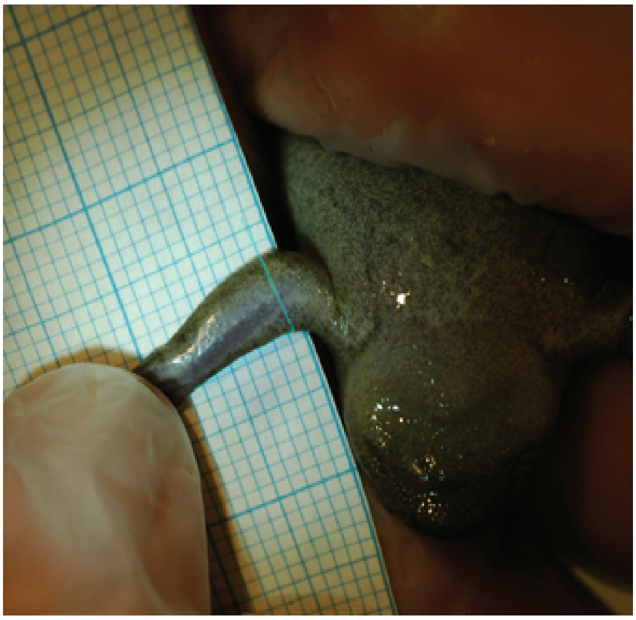

Before the second boosting injection, each male frog was photographed. A piece of graph paper was held beneath the forearm. The camera (Nicon D70, objective AF micro Nikkor 60 mm 1:2:8D) was held by a camera support, approximately 30 cm over a bench, facing it, and a torch was used to illuminate the arm. The forelimb length and width of the male frogs were measured in the photos using an image analysis program, ImageJ. The graph paper beneath the forelimb on the picture was used as a scale. The length was measured on the inside of the forearm, from the bend at the elbow, down to the wrist. The arm width was measured by rotating this drawn line 90°, then moving it to where the “length line” (first measured line described above) ended at the elbow end, and the width was measured at this point.

Figure S2. Measurement of nuptial pad size and colour.

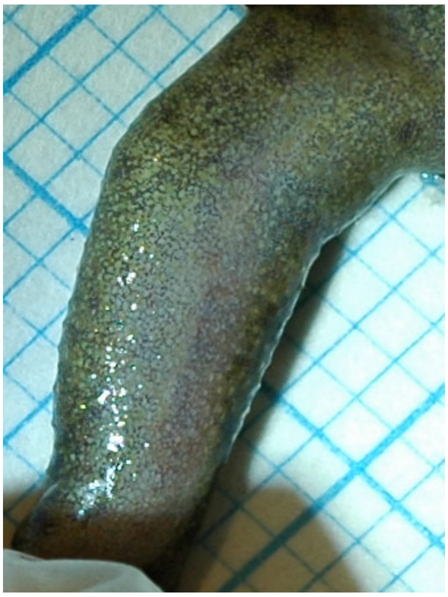

The same photograph was used for forelimb width and nuptial pad measurements. The size and colour intensity of the nuptial pad were analysed using Adobe Photoshop CS6. The nuptial pad was selected using the Quick Selection Tool, and the area (in number of pixels) and colour intensities were recorded. Colour intensity ranged from 0 (black) to 255 (white). Reflections on the arm from water drops have an intense white colour which interferes with the colour intensity measurements. To eliminate this artefact the reflections were removed using the Spot Healing Brush Tool. This tool allows the removal of the reflections by replacing these areas with a composite colour sampled from the skin surrounding the reflection. For each photo the area was calculated by comparing the number of pixels in the selected part with the number of pixels in a 1x2 mm selected area in the graph paper. Each photograph was analysed twice without knowledge of treatment and the mean of these measurements was used for data analysis.

Table S2 - Results of the water chemistry (µg/L). LOD = limit of detection. *N* = total number of samples analysed (3 tanks per treatment).

			Water Change (75%)			
			After		Before	
	LOD	Target	*N*	Mean(SE)	*N*	Mean(SE)
Linuron Low	0.02	9.5	3	7(0.3)	9	7.4(0.8)
Linuron High	0.02	45	3	42(1.7)	9	45(5.3)
Flutamide	5	50	15	45(0.5)	15	29(2)

Figure S3. Mortality in tadpoles, juveniles, adult males and adult females. No differences between groups was observed (mean ± S.E.). Starting *n* numbers were: tadpoles - all treatment groups = 228; juveniles - control & linuron high = 96, flutamide = 64, linuron low = 91; adult males - control = 51, linuron low = 48, linuron high = 55, flutamide = 23; adult females - control = 30, linuron low =38, linuron high = 30, flutamide = 24

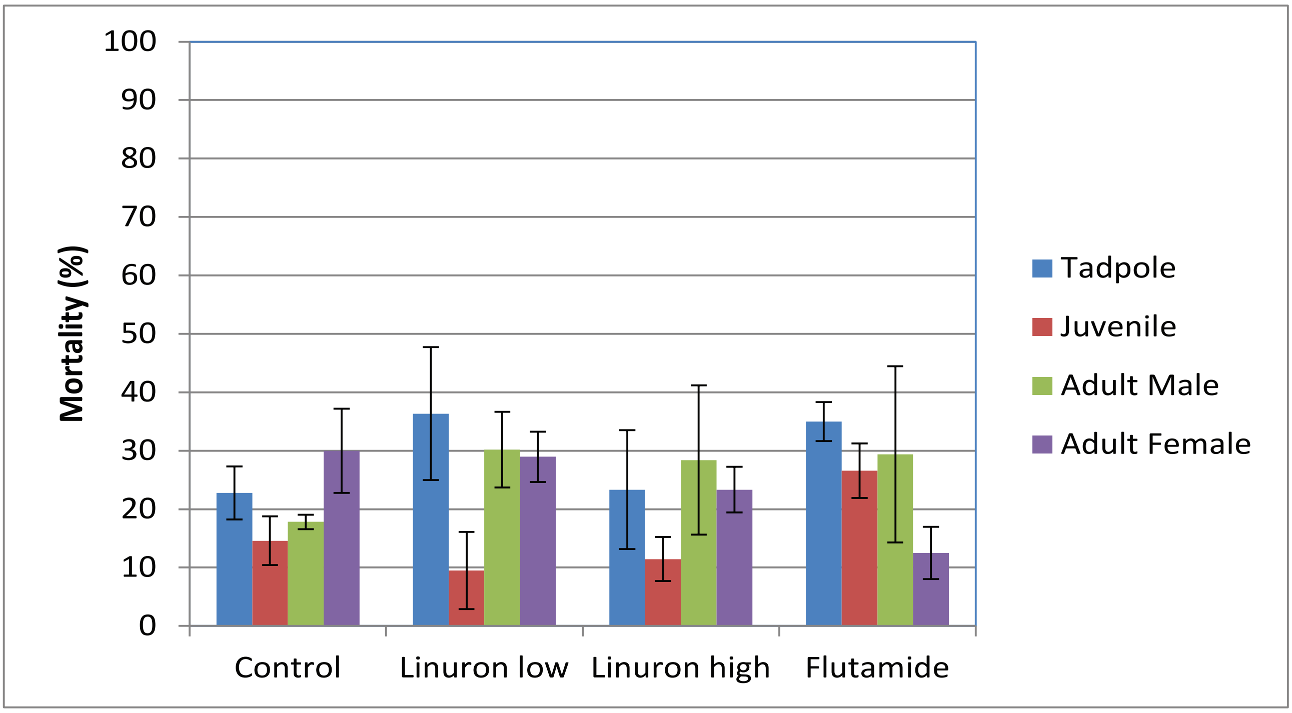

Figure S4. Morphology of metamorphs. No differences between groups were observed (mean ± S.E.) except for hindlimb length across all groups compared to the control (ANCOVA, $p < 0.001$).

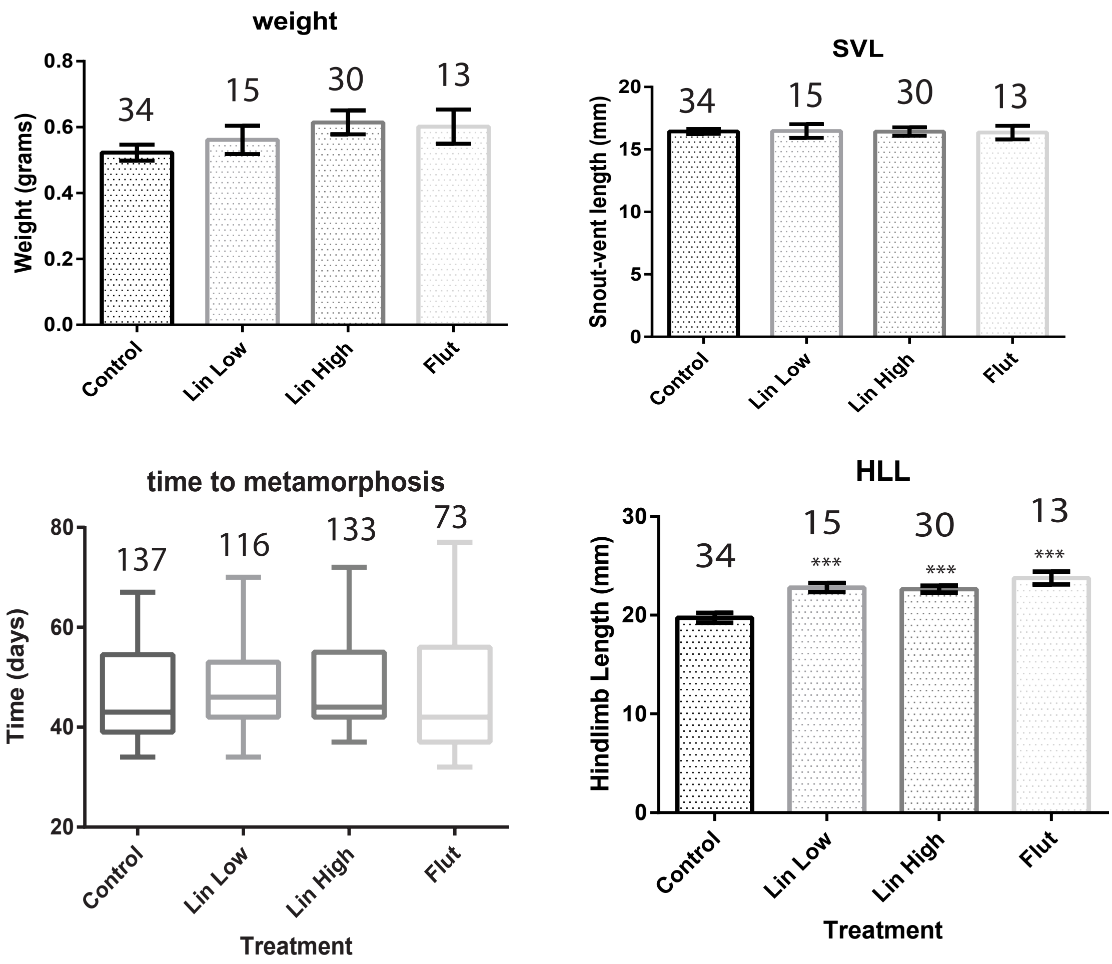

Figure S5. Body weights and gonado-somatic index (GSI) in adult males. No differences between groups were observed (mean ± S.E.).

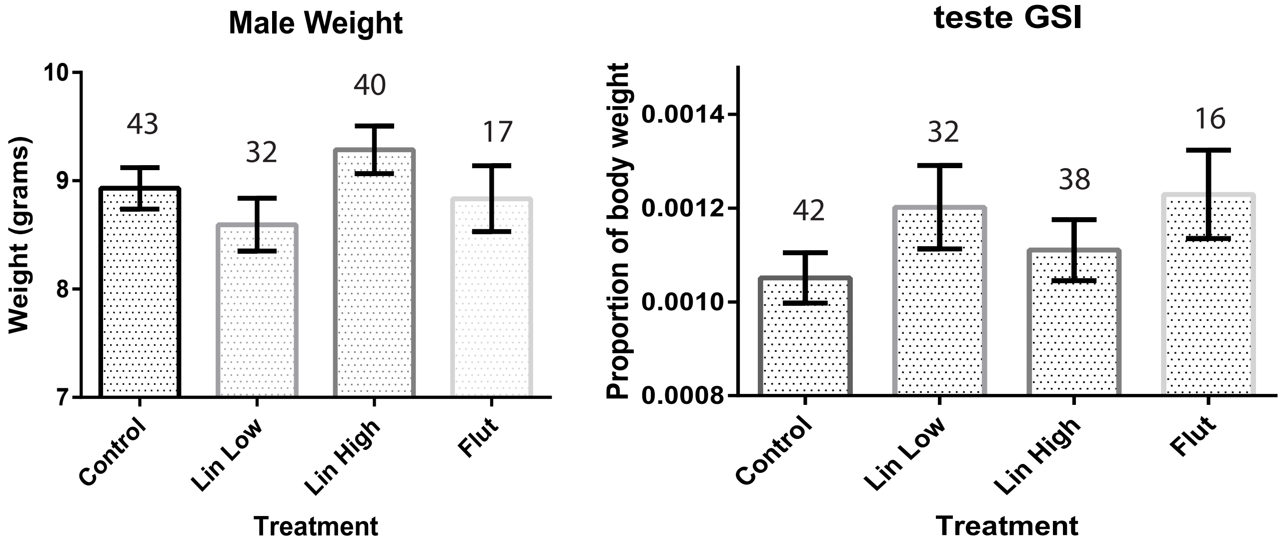

Figure S6. Body weight, gonado-somatic index (GSI) and oviduct weight in adult females (pre-breeding, left and post-breeding, right). Mean ± S.E., significant difference = *

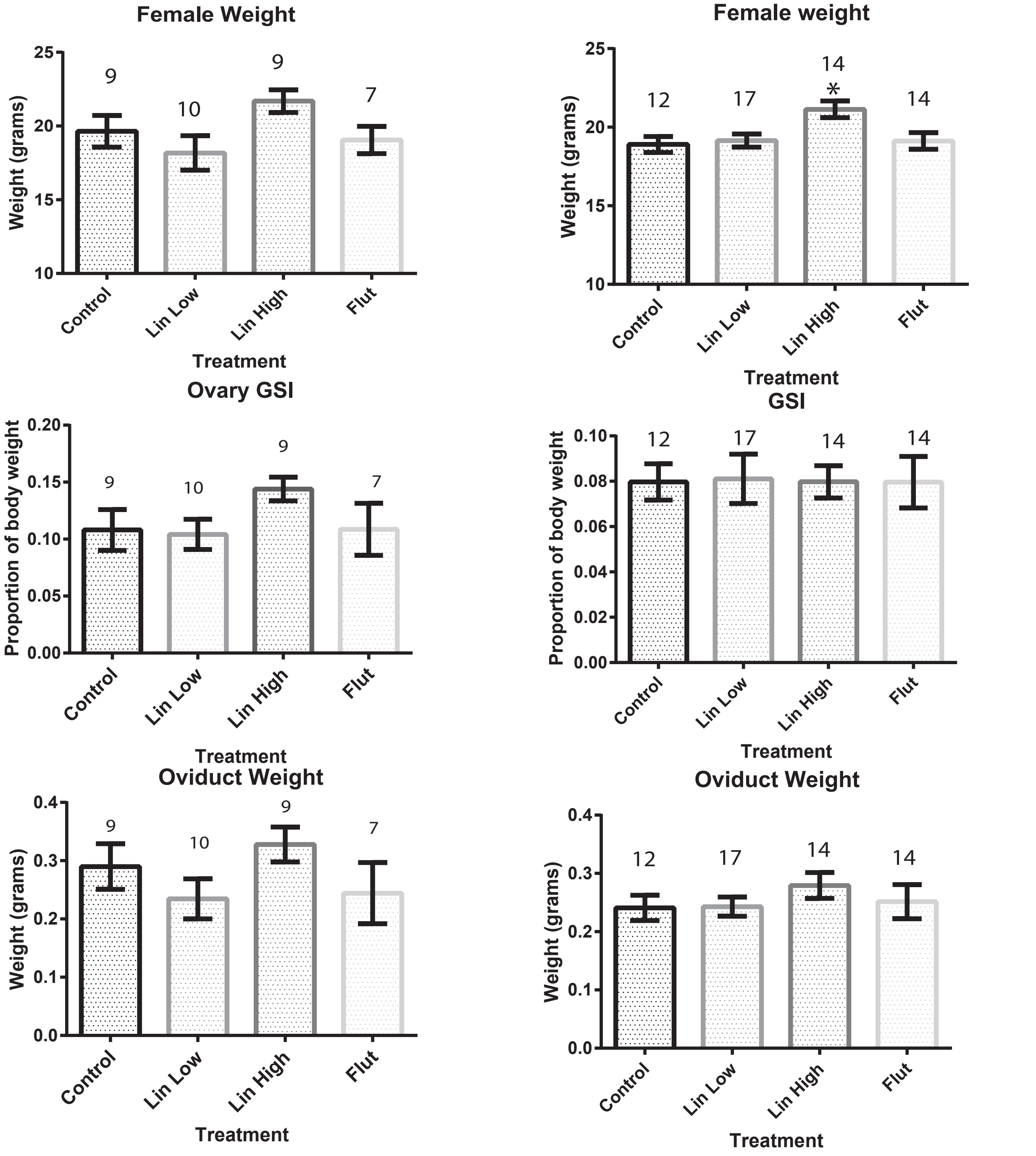

Figure S7. Photomicrograph of gonad sections from juvenile *Xenopus tropicalis* (stage 66) showing (A) ovary containing oocytes in different developmental stages; (B) testis in testicular maturation stage 1- primary spermatogonia without tubules; (C) testes in testicular maturation stage 2- secondary spermatogonia without tubules; (D) testes in testicular maturation stage 3- secondary spermatogonia with tubules (spermatogonia, SPG, spermatocytes, SC, spermatids, ST, and spermatozoa, SZ).

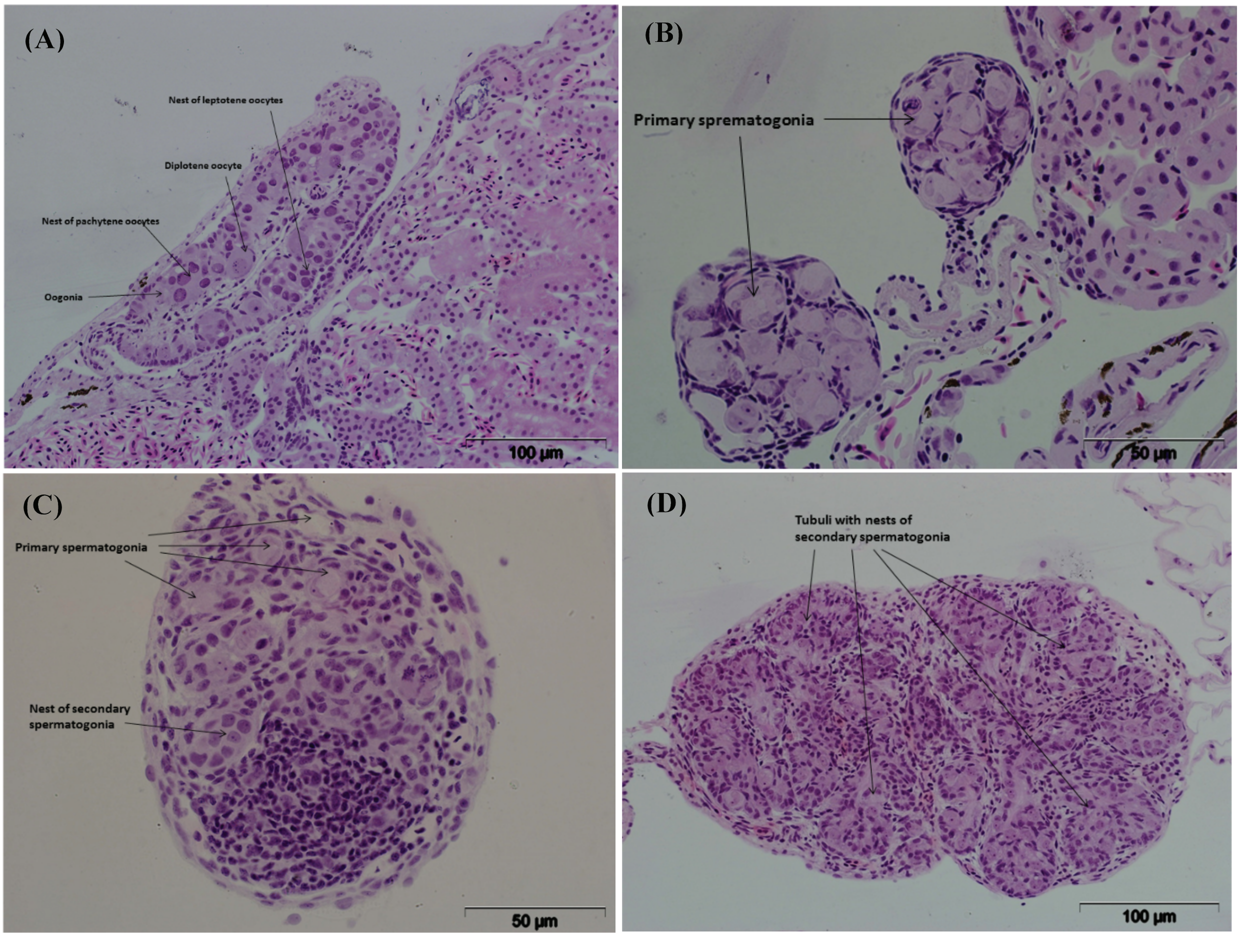

Figure S8. Photomicrographs of gonadal sections from adult *Xenopus tropicalis* showing (A) ovarian oocytes in various developmental stages, (B) a seminiferous tubule with the luminal space filled with spermatozoa (SZ) (Score 3), (C) a seminiferous tubule with spermatozoa occupying approximately half of the luminal space (Score 2), and (D) a seminiferous tubule lacking luminal spermatozoa (Score 1). SPG = spermatogonia, SC = spermatocytes, ST = spermatids, L = lumen.

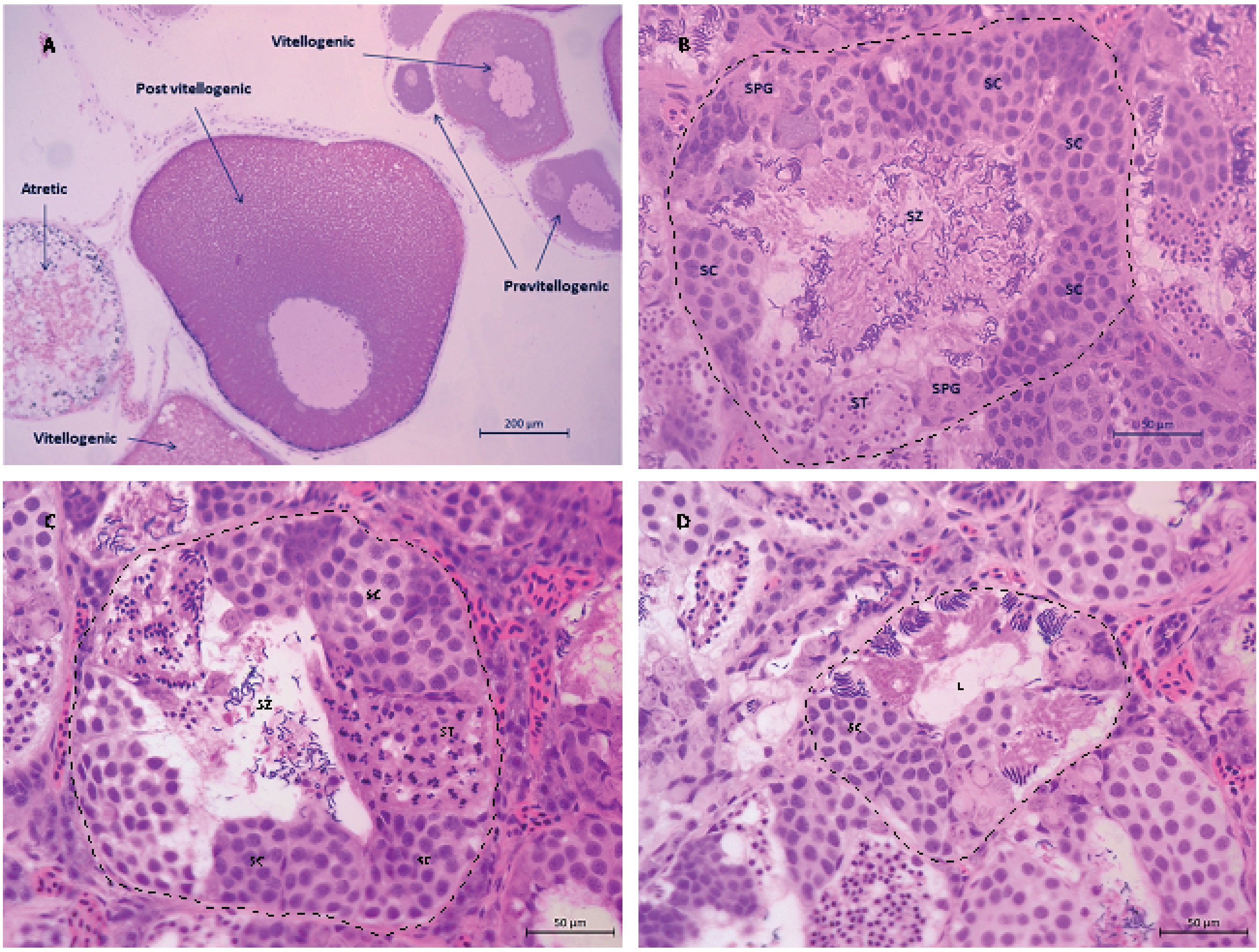

Table S3. Histomorphometrical evaluation of seminiferous tubules in testis form adult male *Xenopus tropicalis* after developmental exposure to linuron (LIN) or flutamide (FLU). Data is presented as mean (S.D.)

Treatment	Cell nests/seminiferous tubule (No.)	Germ cell nest stage (%)			Spermatogonia/ seminiferous tubule (No.)	Size of spermatocytes nests[a]	Spermatozoa in lumen[b]
		Spermatocytes	Spermatid	Spermatozoa			
Control (n=10)	7.0 (1.3)	63 (12)	11 (5)	26 (9)	10 (3)	1.9 (0.6)	1.8 (0.4)
LIN LOW (n=10)	7.8 (2.4)	64 (11)	13 (8)	23 (10)	**19 (6)***	2.1 (0.6)	1.9 (0.6)
LIN HIGH (n=10)	8.4 (2.4)	62 (12)	15 (8)	23 (7)	15 (3)	1.7 (0.5)	1.7 (0.5)
FLU (n=10)	8.2 (0.9)	70 (7)	12 (4)	18 (6)	**20 (8)****	2.2 (4)	1.8 (0.4)

[a] The size of the largest spermatocyte-nest in each seminiferous tubule was determined by using score numbers one to three.

[b]The number of spermatozoa in the seminiferous tubular lumen was estimated using score numbers one to three.

* Significantly different from control (*$p < 0.05$, ** $p < 0.01$), one-way ANOVA and Holm-Sidak .

Table S4. Histomorphometrical evaluation of testes form adult male *Xenopus tropicalis* after developmental exposure to linuron (LIN) or flutamide (FLU).

Treatment	Testis diameter (mm)	Seminiferous tubule diameter (mm)	Seminiferous tubule/testis (No.)	Number of Seminiferous tubule/testis diameter (%)	Testis shape	
					Round %	Irregular %
Control (n=10)	2.8 (0.4)	0.16 (0.02)	186 (61)	67 (19)	70	30
LIN LOW (n=10)	2.7 (0.8)	0.19 (0.03)	131 (66)	52 (26)	60	40
LIN HIGH (n=10)	2.5 (0.6)	**0.22 (0.10)***	131 (66)	51 (20)	50	50
FLU (n=10)	3.2 (0.6)	0.18 (0.02)	173 (39)	54 (7)	70	30

Significantly different from control ($p < 0.05$), Kruskal-Wallis test with Dunn's Multiple Comparison Test.

Figure S9. Photomicrographs of arm sections from adult *Xenopus tropicalis* showing (A) an arm showing many keratinised hooks (KH) and breeding glands (BG) at 25% (x100), (B) an arm showing a BG and KH at 50% (x400), (C) an arm showing normal mucous grands (MG) and no KH at 75% (x200), and (D), an arm showing a NG at 50% (x400).

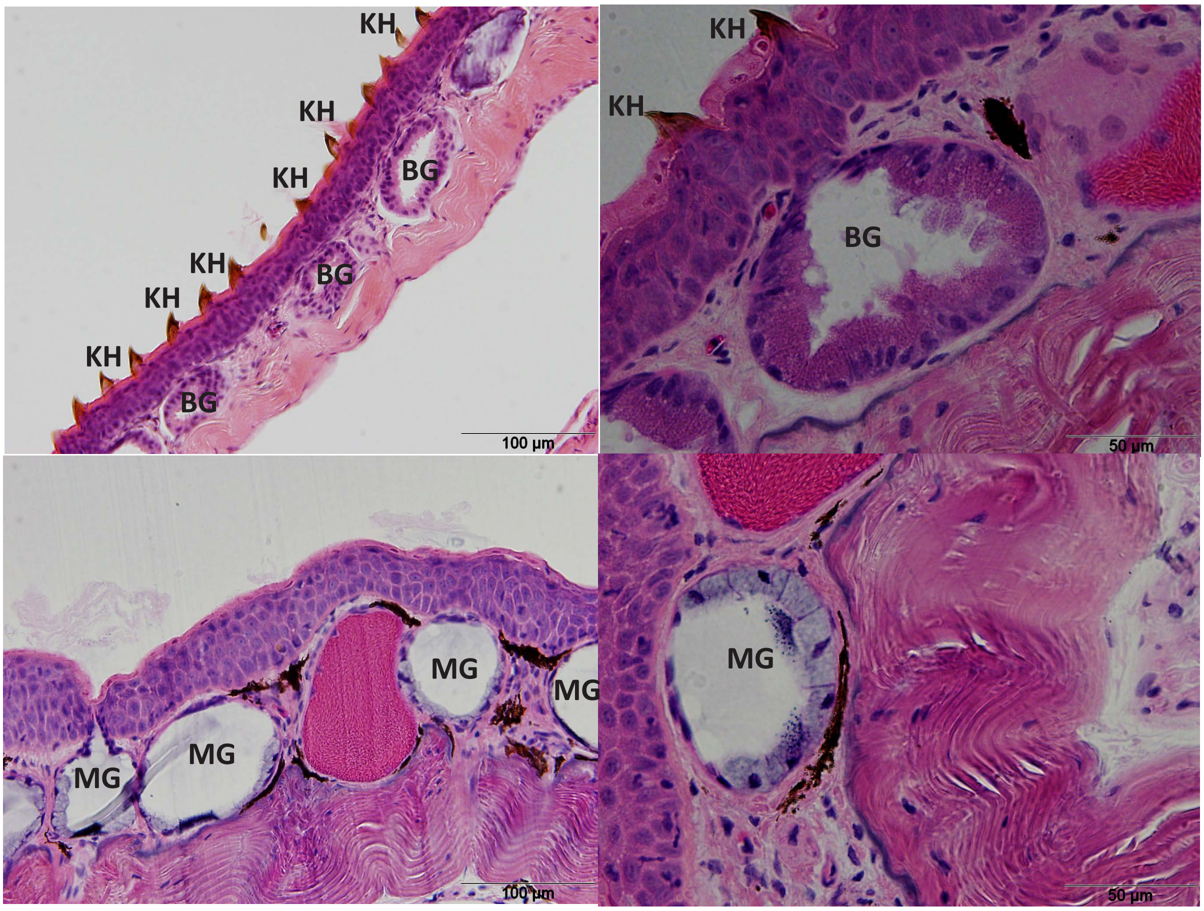

Figure S10. Gene expression in tadpole brains in control organisms. Males (filled symbols) and females (open symbols) did not differ in expression of genes. *ar* expression (bold) increased during ontogeny in both males (black) and in females (grey). *Ar* and *foxl2* expression was related in males (Pearsons', $p < 0.001$). Expression of cyp17, ar or foxl2 (NB: dmrt1, amh and cyp19 were not tested) were not sexually dimorphic. Expression of foxl2 was correlated with ar in males ($p < 0.001$; R2 = 0.70). Expression of ar in the brain increased during ontogeny in males and females, but no changes were seen for cyp17 or foxl2.

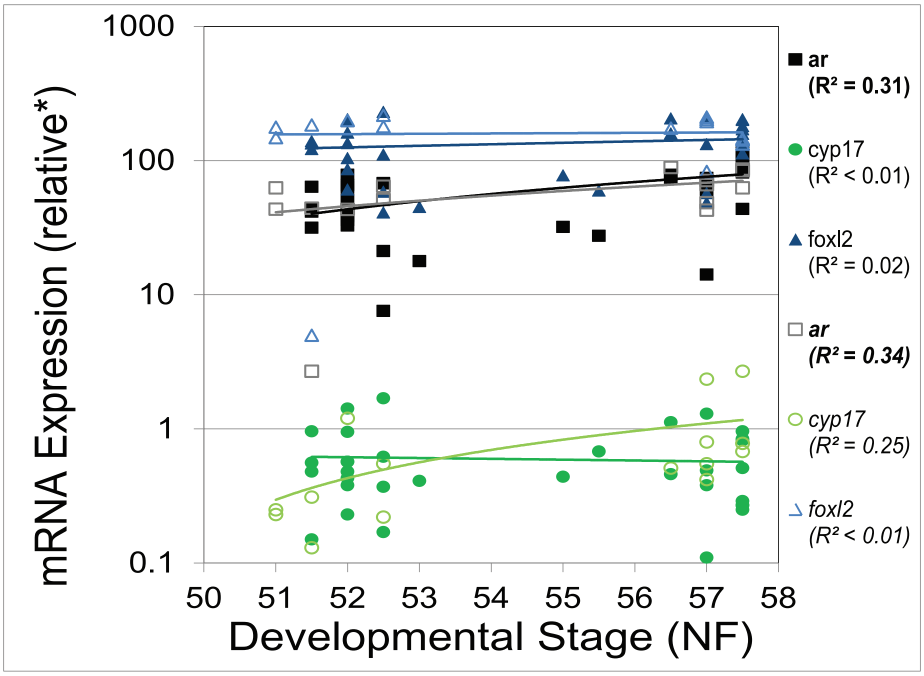

	Male		Female	
	foxl2	***cyp17***	***foxl2***	***cyp17***
ar	0.7***	0.08	0.47	0.32
foxl2	-	0.29	-	0.009

Table S5 - Effects of treatment on gene expression (MANCOVA), *p* values compared to control (Holm-Sidak)

GONAD						
**Male, 51-53**						
Gene	Control	Lin. Low	Lin. High	Flutamide	F Value	ANCOVA *p*
n	17	16	14	11		
dmrt1	2104 ± 463	2185 ± 286	2554 ± 471	2555 ± 373	0.39	0.76
cyp17	2793 ± 444	3367 ± 426	3527 ± 599	4016 ± 723	0.89	0.46
ar	708.7 ± 100	761.4 ±67.5	541.8 ± 37.6	654.3 ± 84.1	1.64	0.2
amh	739.0 ± 144	685.4 ± 97.2	962.6 ± 169	796.4 ± 89.7	0.95	0.46
foxl2	5.855 ± 0.81	5.191 ± 0.79	6.005 ± 1.21	6.456 ± 1.00	0.33	0.81
cyp19	0.556 ± 0.11	0.545 ± 0.18	0.498 ± 0.09	0.667 ± 0.16	0.3	0.83
					lambda = 1.3	0.2
**Female, 51-53**						
Gene	Control	Lin. Low	Lin. High	Flutamide	F Value	ANCOVA *p*
n	7	8	9	5		
dmrt1	391.8 ± 60.5	446.2 ± 77.0	547.6 ± 72.5	478.8 ± 126	1.46	0.27
cyp17	2193 ± 480	1632 ± 207	1697 ± 291	1520 ± 234	0.64	0.6
ar	674.9 ± 118	856.1 ± 97.2	515.3 ± 62.0	908.4 ± 186	5.21	**0.01**
amh	209.3 ± 13.3	226.0 ± 20.8	192.9 ± 10.5	249.9 ± 19.0	1.71	0.21
foxl2	44.96 ± 11.1	**87.75 ± 17.2#**	59.24 ± 12.6	**107.9 ± 23.5****	3.23	**0.05**
cyp19	8.632 ± 2.14	13.06 ± 2.74	11.79 ± 2.71	19.17 ± 4.45	1.76	0.2
					lambda = 1.96	**0.05**

GONAD						
**Male, 55-58**						
Gene	Control	Lin. Low	Lin. High	Flutamide	F Value	ANCOVA *p*
n	16	6	19	7		
dmrt1	9675 ± 738	13138 ± 1906	9891 ± 769	9963 ± 1393	1.66	0.2
cyp17	19604 ± 1599	28412 ± 6735	19727 ± 2100	20791 ± 3573	1.28	0.3
ar	1934 ± 198	2371 ± 310	1737 ± 165	1909 ± 300	0.99	0.41
amh	2624 ± 291	**3925 ± 592#**	2419 ± 180	3258 ± 385	3.49	**0.03**
foxl2	8.363 ± 1.05	14.92 ± 3.29	13.50 ± 4.33	12.26 ± 3.07	0.45	0.72
cyp19	0.885 ± 0.15	0.924 ± 0.41	0.863 ± 0.19	0.809 ± 0.18	0.02	0.99
					lambda = 0.88	0.6
**Female, 55-58**						
Gene	Control	Lin. Low	Lin. High	Flutamide	F Value	ANCOVA *p*
n	8	10	5	9		
dmrt1	1640 ± 540	1715 ± 311	1451 ± 487	3062 ± 891	0.58	0.64
cyp17	1286 ± 582	867.3 ± 142	940.7 ± 171	712.4 ± 54.6	0.66	0.59
ar	1024 ± 192	1236 ± 150	1301 ± 176	1366 ± 119	0.76	0.53
amh	485.8 ± 175	335.7 ± 13.9	302.0 ± 42.6	353.0 ± 38.5	0.49	0.7
foxl2	188.7 ± 21.2	249.2 ± 36.1	207.0 ± 30.7	260.3 ± 37.5	0.88	0.47
cyp19	17.94 ± 5.23	13.41 ± 1.67	12.81 ± 1.42	12.35 ± 1.40	0.53	0.67
					lambda = 0.67	0.8

P values compared to control: # p = 0.06, * < 0.05, ** < 0.01 (Holm-Sidak)

Table S6 Effects of treatment on gene expression (MANCOVA), *p* values compared to control (Holm-Sidak)

BRAIN						
**Male, 51-53**						
Gene	Control	Lin. Low	Lin. High	Flutamide	F Value	ANCOVA *p*
n	17	15	14	11		
cyp17	0.41 ± 0.14	0.489 ± 0.07	0.453 ± 0.08	0.405 ± 0.09	0.99	0.4
ar	45.21 ± 4.90	48.66 ± 5.36	39.67 ± 6.87	41.15 ± 5.90	0.44	0.73
foxl2	127.2 ± 14.1	140.3 ± 15.6	92.57 ± 17.0	126.0 ± 13.9	1.64	0.20
					lambda = 1.0	0.44
**Female, 51-53**						
Gene	Control	Lin. Low	Lin. High	Flutamide	F Value	ANCOVA *p*
n	7	8	9	5		
cyp17	0.61 ± 0.10	0.609 ± 0.14	0.419 ± 0.07	0.456 ± 0.12	1.86	0.17
ar	44.54 ± 7.74	53.34 ± 8.90	50.58 ± 8.08	56.58 ± 9.02	0.26	0.84
foxl2	158.6 ± 26.8	187.6 ± 49.3	201.3 ± 49.3	146.0 ± 17.9	0.4	0.78
					lambda = 1.69	0.16

BRAIN						
**Male, 55-58**						
Gene	Control	Lin. Low	Lin. High	Flutamide	F Value	ANCOVA *p*
n	16	6	19	7		
cyp17	0.574 ± 0.09	0.613 ± 0.09	0.507 ± 0.07	0.531 ± 0.19	0.13	0.94
ar	73.94 ± 7.87	88.24 ± 7.24	72.88 ± 2.77	53.63 ± 8.63	3.92	**0.03**
foxl2	140.8 ± 14.0	179.9 ± 21.0	151.6 ± 14.0	98.13 ± 26.0	3.56	**0.04**
					lambda = 1.53	0.14
**Female, 55-58**						
Gene	Control	Lin. Low	Lin. High	Flutamide	F Value	ANCOVA *p*
n	8		5	9		
cyp17	1.098 ± 0.32	0.841 ± 0.18	0.672 ± 0.19	0.762 ± 0.22	0.57	0.58
ar	68.87 ± 1.10	76.21 ± 6.36	72.80 ± 11.5	66.21 ± 8.15	0.16	0.91
foxl2	160.7 ± 15.2	164.6 ± 18.5	160.3 ± 42.2	169.0 ± 28.5	0.17	0.95
					lambda = 0.75	0.80

Figure S11 Gene expression in tadpole brains in males (filled symbols) and females (open symbols) Bold in the figure legend indicates significant increase in expression during ontogeny for *foxl2* and *ar* (*cyp17* expression was very low and did not differ from expression observed in controls (Fig. S9)).

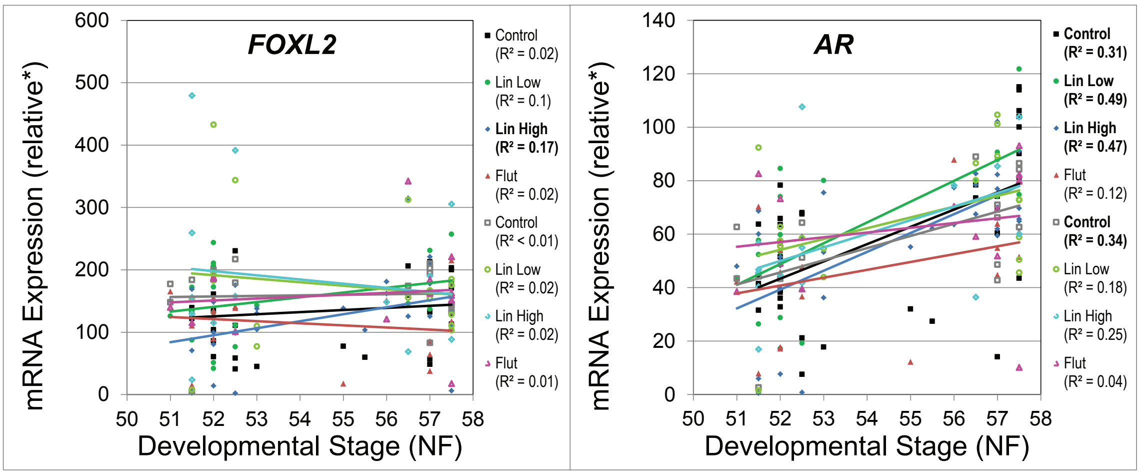

		Male		Female	
		foxl2	***cyp17***	***foxl2***	***cyp17***
**Control**	***ar***	0.7***	0.08	0.47	0.32
	foxl2	-	0.29	-	0.009
**Lin Low**	***ar***	0.7***	0.16	0.34	0.25
	foxl2	-	0.09	-	0.29
**Lin High**	***ar***	0.76***	0.19	0.55*	0.32
	foxl2	-	0.34*	-	-0.07
**Flut**	***ar***	0.39	0.52*	0.44	-0.06
	foxl2	-	0.33	-	0.15

Table S7. Effects of treatment on the relationship between expressed target genes in males and females.

In both males and females, the interrelationships between the expressed genes differed in response to treatments, compared with their respective controls (shown in Figure 3). In response to linuron low/high treatments in males *foxl2* was correlated with *dmrt1/amh* ($p < 0.01$; $R2 > 0.43$) that was not observed in the controls (Figure 3). Significant positive correlations see in control females (*dmrt1 v. cyp17/amh/cyp19*; *ar v. foxl2; amh v. cyp19*; Figure 3) were not observed in the linuron low or flutamide treatment groups ($p < 0.05$; $R2 < 0.67$).

	Male (r^2 values)					Female (r^2 values)				
**Lin low**	*cyp17*	*amh*	*ar*	*foxl2*	*cyp19*	*cyp17*	*amh*	*ar*	*foxl2*	*cyp19*
dmrt1	0.87***	0.92***	0.64***	**0.55**↑**	0.35	**-0.22↓**	**0.49*↓**	0.73***	0.69**	**0.09↓**
cyp17	-	0.88***	0.51***	0.56**	0.36	-	-0.37	-0.4	-0.18	0.28
amh		-	0.65**	0.61**	0.32		-	0.26	0.63**	**0.25↓**
ar			-	0.64**	0.16			-	**0.31*↓**	0.11
foxl2				-	0.22				-	0.69**
**Lin high**	*cyp17*	*amh*	*ar*	*foxl2*	*cyp19*	*cyp17*	*amh*	*ar*	*foxl2*	*cyp19*
dmrt1	0.89***	0.91***	0.83***	0.55***	0.27	0.26	**0.71**↓**	0.75**	0.59*	0.51
cyp17	-	0.89***	0.67***	0.43**	0.15	-	-0.06	-0.12	-0.08	0.61*
amh		-	0.53***	**0.43**↑**	0.26		-	0.79***	0.81***	**0.43↓**
ar			-	0.37*	0.27			-	0.81***	0.51
foxl2				-	**0.65***↑**				-	0.58*
**Flut**	*cyp17*	*amh*	*ar*	*foxl2*	*cyp19*	*cyp17*	*amh*	*ar*	*foxl2*	*cyp19*
dmrt1	0.91***	0.90***	0.84***	0.43	0.08	**-0.68**↓**	**0.67**↓**	0.60*	-0.44	**-0.17↓**
cyp17	-	0.92***	0.83***	0.52*	0.29	-	-0.25	-0.43	0.20	0.43
amh		-	0.90***	0.33	0.17		-	0.32*	0.55*	**0.06↓**
ar			-	0.46*	0.04			-	**0.50**↓**	0.37
foxl2				-	0.59*				-	0.08

Asterices in dicate significant corr elations between analysed genes (normal text, no difference compared to control) or significant differences in the linear regression compared to the control (bold text [ANCOVA, with stage as a covariate]) with the arrows indicating the direction of the change. *$p < 0.05$, **$p < 0.01$, ***$p < 0.001$.

Table S8. Effects of treatment on brain/testis *ar* and arm/testis *gr*.

**Brain *ar***	**Mean(SE)**	**Estimate**	**Error**	***P* value**
Control	0.78(0.006)	0.83	0.06	-
Linuron Low	0.77(0.006)	-0.01	0.02	0.45
Linuron High	0.81(0.013)	0.02	0.02	0.23
Flutamide	0.79(0.012)	0.015	0.03	0.57
Male Weight	*factor*	-0.002	0.006	0.79
Interval	*factor*	-0.001	0.001	0.16
Model				*0.3*
**Testis *ar***	**Mean(SE)**	**Estimate**	**Error**	***P* value**
Control	0.88(0.01)	0.88	0.09	-
Linuron Low	0.86(0.02)	-0.01	0.03	0.71
Linuron High	0.84(0.01)	-0.04	0.03	0.27
Flutamide	0.84(0.02)	-0.04	0.04	0.38
Male Weight	*factor*	-0.007	0.01	0.94
Interval	*factor*	< 0.001	0.01	096
Model				*0.9*
**Arm *gr***	**Mean(SE)**	**Estimate**	**Error**	***P* value**
Control	0.86(0.01)	0.99	0.08	-
Linuron Low	0.88(0.01)	0.01	0.03	0.75
Linuron High	0.88(0.01)	0.01	0.03	0.55
Flutamide	0.89(0.02)	0.04	0.03	0.22
Male Weight	*Factor*	-0.01	0.01	0.47
Interval	*Factor*	0.003	0.001	**0.01**
Model				*0.16*
**Testis *gr***	**Mean(SE)**	**Estimate**	**Error**	***P value***
Control	0.99(0.01)	0.95	0.09	-
Linuron Low	0.94(0.02)	-0.05	0.03	0.11
Linuron High	0.95(0.01)	-0.04	0.03	0.25
Flutamide	0.98(0.01)	-0.01	0.04	0.73
Male Weight	*factor*	<0.001	0.01	0.78
Interval	*factor*	<0.001	0.001	0.75
Model				*0.6*

Table S9 Interaction of the human androgen response element with upstream promotor regions of various genes.

	Species	Score	Relative score	Start	End	Strand	sequence
dmrt1	*X.tropicalis*	11.889	0.90	177	191	1	AGGCACAGTAAGTAA
cyp17	*X.tropicalis*	11.733	0.90	1410	1424	1	GGGTACAAACTGCAC
amh	*X.tropicalis*	12.028	0.90	3258	3272	-1	AAGTACACAGAGGCC
foxl2	*X.tropicalis*	12.412	0.91	860	874	-1	AAGAACATTGAGAAA
sox9	*X.laevis*	9.622	0.86	151	165	-1	CGGCACAGACAGGGC
sox9	*H.sapiens*	12.851	0.92	2659	2673	1	AGGGACACACTGCCC
rspl8	*X.tropicalis*	9.990	0.88	2180	2194	-1	GAGAACAGATTCTAA

Scores were calculated using the JASPAR online database (http://jaspar.genereg.net/) which is a transcription factor binding profile database. The upstream promotor region (4000 bp) for each gene was found using the BLAST database, and this sequence was inserted into the JASPAR database to search for similarity with the human androgen response element sequence with a threshold of 80%.
